# Interplay Among Reward Processing, Schizotypal Traits, and Psychosocial Stress in a Large Chinese Young Adult Sample: A Cross‐Sectional Network Analysis

**DOI:** 10.1002/pchj.70102

**Published:** 2026-05-20

**Authors:** Donghao Tuo, Yi‐hang Huang, Simon S. Y. Liu, Jia Huang, Raymond C. K. Chan

**Affiliations:** ^1^ Neuropsychology and Applied Cognitive Neuroscience Laboratory, State Key Laboratory of Cognitive Science and Mental Health, Institute of Psychology Chinese Academy of Sciences Beijing China; ^2^ Department of Psychology University of Chinese Academy of Sciences Beijing China; ^3^ Department of Psychiatry, School of Clinical Medicine The University of Hong Kong Hong Kong Hong Kong

**Keywords:** effort‐reward imbalance, network analysis, pleasure experience, reward motivation, reward sensitivity, schizotypal traits

## Abstract

Reward processing comprises reward sensitivity, motivation, and pleasure experience, but how these components jointly relate to schizotypal traits and psychosocial stress in young adult samples remains unclear. Using network analysis in a large Chinese young adult sample (*N =* 6814), we examined multivariate associations among reward sensitivity (BAS), schizotypal traits (MSS), reward motivation (MAP‐SR), pleasure experience (TEPS), and psychosocial stress (ERI). The results indicated that BAS‐Fun seeking showed the highest strength centrality and expected influence. MSS‐Disorganized schizotypal traits served as a bridge node between reward sensitivity and reward motivation and pleasure experience. Subsequent mediation analysis also supported these findings. Network comparison tests indicated no difference in global strength between the high‐ and low‐psychosocial‐stress groups, but a significant difference in overall network structure. These findings highlight impulsive Fun seeking and disorganized schizotypal traits as potentially informative targets for mechanistic and intervention‐oriented research across the psychosis continuum in young adult cohorts.

## 1 Introduction

Reward processing refers to neural and psychological mechanisms supporting motivated behavior and is often decomposed into reward sensitivity, motivation, and pleasure experience (Berridge and Robinson 1998; Kring and Barch 2014; Strauss et al. 2014). Alterations in reward processing relate to anhedonia, motivational deficits, and functional impairment across the psychosis spectrum, including associations with schizotypal traits (Barch et al. 2014; Kring and Barch 2014; Strauss et al. 2014; Wang et al. 2023). Research in schizophrenia frequently emphasizes asymmetric deficits, with relatively preserved consummatory pleasure but reduced anticipatory pleasure and goal‐directed engagement (Gard et al. 2007; Visser et al. 2020). Young adulthood is the typical period for the onset of psychosis spectrum disorders (Solmi et al. 2022). However, evidence regarding this period varies across samples, and this complexity is compounded by literature largely organized around single constructs or task domains (Strauss et al. 2014; Wang et al. 2023). Consequently, the multivariate relationships among reward sensitivity, motivation, and pleasure experience remain poorly characterized, particularly, in nonclinical young adult samples.

A psychosis continuum perspective extends reward processing research to nonclinical samples to characterize vulnerability‐related traits in the general population (DeRosse and Karlsgodt 2015; Staines et al. 2022; Harju‐Seppänen et al. 2022; Carruzzo et al. 2023). Schizotypal traits are often viewed as a latent vulnerability for schizophrenia‐related phenotypes and show a multidimensional structure, typically comprising positive, negative, and disorganized dimensions, corresponding to different forms of functional vulnerability (Kwapil and Barrantes‐Vidal 2015; Tiego et al. 2023; Hernández et al. 2023; Kemp et al. 2025). Negative schizotypal traits are associated with reduced approach motivation, avolition, and anhedonia, particularly social anhedonia, and have been linked to reduced reward motivation and effort allocation for reward (Kwapil 1998; Wang et al. 2024; Yan et al. 2023; Chu et al. 2023). Positive schizotypal traits are characterized by perceptual aberrations, odd beliefs, and magical thinking, and are associated with aberrant salience attribution and heightened physiological arousal to social stress, as well as neural differences during reward anticipation (Chun et al. 2019; Premkumar et al. 2021; Carruzzo et al. 2023). Disorganized schizotypal traits involve cognitive‐behavioral disorganization and reduced cognitive control. These traits may impede the conversion of cue‐elicited approach into sustained goal‐directed action and may manifest as state‐like disorganization under heightened stress reactivity, thereby contributing to the decoupling of reward components (Steffens et al. 2018; Tiego et al. 2023; Kemp et al. 2025; Rónai et al. 2026).

As the expression of schizotypal vulnerability is influenced by environmental context, psychosocial stress may help explain why different schizotypal dimensions show different patterns under stress (Hernández et al. 2023; Rónai et al. 2026). The diathesis‐stress model links psychosis‐spectrum risk to environmental stress exposure and highlights hypothalamic–pituitary–adrenal axis activation and dopaminergic modulation as key amplifiers of vulnerability‐related emotional and behavioral phenotypes (Walker and Diforio 1997; Pruessner et al. 2017). The effort‐reward imbalance (ERI) model operationalizes chronic psychosocial stress through sustained ERI and overcommitment as a high‐investment coping tendency (Siegrist 1996; Siegrist et al. 2004; Siegrist and Li 2016; Tsutsumi and Kawakami 2004). ERI shows close links to reward motivation and functional abnormalities (Pan et al. 2023; Yan et al. 2021, 2023). Daily‐life studies further indicate dimension‐specific stress reactivity across schizotypal traits; disorganized traits predict stronger stress reactivity of psychotic‐like experiences, more negative appraisal after stressful events, and affective dysregulation (Hernández et al. 2023; Rónai et al. 2026). Longitudinal evidence also supports joint pathways from psychosocial stress and schizotypal traits to reward processing; baseline ERI predicts subsequent decreases in reward motivation and relates to schizotypal dimensions and anticipatory pleasure (Pan et al. 2023). Despite these links, existing work often tests single variables or narrow pathways (Pan et al. 2023; Yan et al. 2021, 2023; Strauss et al. 2014), and comparative evidence on the multivariate association structure linking reward‐processing components and schizotypal‐trait dimensions across stress levels remains limited.

Reward‐processing components, schizotypal‐trait dimensions, and psychosocial stress levels may form an interdependent multivariate association structure. Traditional approaches such as structural equation modeling usually depend on prespecified paths or latent structures and mainly test hypothesized relations, limiting direct characterization of conditional dependencies among multiple components in a single model (Christ et al. 2014; Briganti et al. 2024; Borsboom et al. 2021). Network analysis targets this goal by estimating partial associations among multiple observed variables, treating variables as nodes and conditional associations as edges (Epskamp et al. 2018). Centrality indices quantify node prominence, including bridge centrality for cross‐community connectivity (Epskamp et al. 2018; Jones et al. 2021), and network comparison tests (NCTs) evaluate group differences in global structure, global strength, and edge‐specific effects (van Borkulo et al. 2023).

The present study used self‐report measures to quantify reward‐processing components, multidimensional schizotypal traits, and psychosocial stress in a large Chinese community sample, and estimated a network with scale dimensions as nodes. Based on prior work (Pan et al. 2023; Dodell‐Feder et al. 2019; Kemp et al. 2025; Rónai et al. 2026), we hypothesized that reward sensitivity would show strong connectivity with reward motivation and pleasure experience. We also hypothesized that disorganized schizotypal traits would show prominent centrality and bridge connectivity with reward motivation and pleasure experience. Finally, we tested whether network structure and key edges differed across groups defined by ERratios.

## 2 Methods

### 2.1 Study Design and Setting

This cross‐sectional observational study followed the STROBE statement (von Elm et al. 2007). Data were collected in Beijing, China, from May 2017 to November 2022. Participants were recruited from universities and local communities via campus advertisements and community outreach. The protocol was approved by the Ethics Committee of the Institute of Psychology, Chinese Academy of Sciences (protocol number H22106). Data were collected for the present research project. A subset of participants (*n =* 1212) contributed to a prior network analysis on childhood trauma and dimensional schizotypy (Huang et al. 2023).

### 2.2 Participants

**FIGURE 1:**
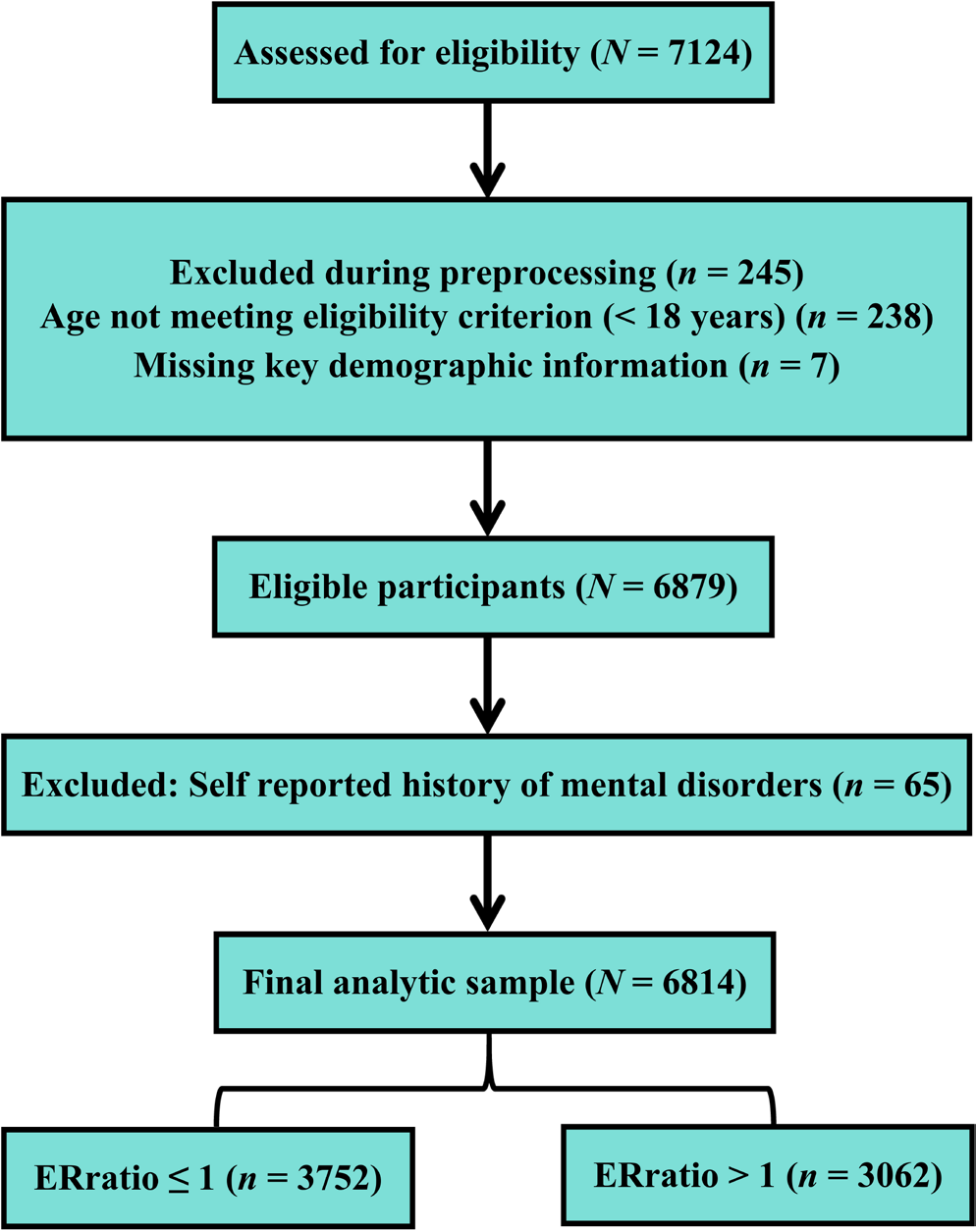
Flowchart of participant eligibility assessment, preprocessing exclusions, final analytic sample, and ERratio‐based group assignment. A total of 7124 individuals were assessed for eligibility; 245 were excluded during preprocessing due to age < 18 years (*n =* 238) or missing key demographic information (*n =* 7). Among 6879 eligible participants, 65 reporting a history of mental disorders were excluded, yielding a final analytic sample of 6814. For subsequent network comparisons, participants were further classified into a low‐psychosocial‐stress group (ERratio ≤ 1; *n =* 3752) and a high‐psychosocial‐stress group (ERratio > 1; *n =* 3062).

A total of 7124 participants completed the survey. Participants were eligible if they were aged 18 years or older. During preprocessing, 245 records were excluded because they did not meet the age eligibility criterion (*n =* 238) or had missing key demographic information (*n =* 7), yielding 6879 eligible participants. We then excluded 65 participants who reported a history of mental disorders, leaving a final analytic sample of 6814 participants. All participants provided written informed consent prior to inclusion. The recruitment and sample selection process is summarized in Figure 1.

### 2.3 Measures

#### 2.3.1 Reward Sensitivity

The Chinese BAS subscale of the BIS/BAS Scale measured reward sensitivity (Carver and White 1994; Li et al. 2008). BAS includes 13 items across BAS‐Fun seeking (4 items), BAS‐Reward Responsiveness (5 items), and BAS‐Drive (4 items), rated on a 4‐point Likert scale. Higher scores indicate greater reward sensitivity. Internal consistency in the present sample was acceptable (BAS‐Fun seeking α = 0.70; BAS‐Reward Responsiveness α = 0.80; BAS‐Drive *α* = 0.76).

#### 2.3.2 Pleasure Experience

The Chinese version of the Temporal Experience of Pleasure Scale (TEPS) (Gard et al. 2006; Chan et al. 2012) assessed anticipatory and consummatory pleasure. The original TEPS contains 18 items rated on a 6‐point Likert scale; the Chinese adaptation introduced minor culturally informed revisions and supported a 19‐item form for psychometric analyses. We computed TEPS‐Anticipatory and TEPS‐Consummatory scores. Items were rated from 1 (*very untrue of me*) to 6 (*very true of me*); higher scores indicate greater capacity for experiencing pleasure. Internal consistency in the present sample was acceptable (TEPS‐Anticipatory *α* = 0.76; TEPS‐Consummatory *α* = 0.82).

#### 2.3.3 Reward Motivation

The Chinese adaptation of the Motivation and Pleasure Scale‐Self Report (MAP‐SR) (Llerena et al. 2013; Wang et al. 2020) was used to index motivation and pleasure in daily life. The 15‐item scale covers pleasure experience and motivational effort across social, recreational or work, close relationship, and motivational effort domains, with items rated from 0 to 4. We used the total score as the primary index; higher scores indicate higher motivation. Internal consistency in the present sample was excellent (*α* = 0.91).

#### 2.3.4 Schizotypal Traits

The Chinese version of the Multidimensional Schizotypy Scale (MSS) (Kwapil et al. 2018; Zou et al. 2020; Wang et al. 2021) was used to assess schizotypal traits. This 77‐item scale measures three dimensions: positive schizotypal traits (26 items), negative schizotypal traits (26 items), and disorganized schizotypal traits (25 items). Participants responded yes/no. Higher scores indicate greater severity of the respective trait dimension. Internal consistency in the present sample was excellent (MSS‐Positive *α* = 0.91; MSS‐Negative *α* = 0.83; MSS‐Disorganized *α* = 0.92).

#### 2.3.5 Psychosocial Stress

A Chinese version of the ERI questionnaire for school settings (Li et al. 2010; Siegrist 1996) was used to measure psychosocial stress. The scale includes ERI‐Effort (5 items), ERI‐Reward (11 items), and ERI‐Overcommitment (3 items), rated on a 5‐point Likert scale. We computed ERratio (the effort‐reward ratio) = Effort/(Reward × *c*), with *c* = 5/11 adjusting for unequal item numbers; ERratio > 1 indicates high psychosocial stress. Internal consistency in the present sample was acceptable (ERI‐Effort *α* = 0.77; ERI‐Reward *α* = 0.82; ERI‐Overcommitment *α* = 0.73).

### 2.4 Network Estimation, Visualization, and Stability

A Gaussian graphical model was estimated in R using qgraph and bootnet (Epskamp et al. 2012, 2018). The network comprised 12 nodes: BAS‐Fun seeking, BAS‐Reward Responsiveness, BAS‐Drive, TEPS‐Anticipatory, TEPS‐Consummatory, MAP‐SR total score, MSS‐Positive, MSS‐Negative, MSS‐Disorganized, ERI‐Effort, ERI‐Reward, and ERI‐Overcommitment. Edges represented EBICglasso‐regularized partial correlations (LASSO with EBIC tuning *γ* = 0.5) (Tibshirani 1996; Friedman et al. 2008; Foygel and Drton 2010; Epskamp and Fried 2018). ERratio served only for subgrouping and was not modeled as a node. Network visualization used the Fruchterman‐Reingold algorithm (Fruchterman and Reingold 1991). We also computed centrality measures, including strength, betweenness, closeness, and expected influence (EI), and node predictability (Haslbeck and Waldorp 2018). Edge‐weight accuracy and centrality stability were examined via bootstrap procedures (1000 iterations) implemented in bootnet (version 1.5.6) (Epskamp et al. 2018).

### 2.5 Network Comparison

Network structure was compared across stress levels using the NCT (van Borkulo et al. 2023) between the high‐stress group (ERratio > 1; *n =* 3062) and the low‐stress group (ERratio ≤ 1; *n =* 3752), consistent with the eligibility assessment, preprocessing exclusions, and ERratio‐based group assignment shown in Figure 1. Global structure invariance, global strength, and edge‐specific differences were evaluated using 10,000 permutations, with Holm‐Bonferroni correction applied to edge‐level tests (Holm 1979).

### 2.6 Mediation Analysis

The mediation models were specified in R using the lavaan package (version 0.6.19) (Rosseel 2012). BAS‐Fun seeking was set as the independent variable, MSS‐Disorganized schizotypal traits as the mediator, and TEPS (TEPS total score) and MAP (MAP‐SR total score) as the dependent variables, respectively. Total, direct, and indirect effects were estimated with 5000 bootstrap samples and 95% bootstrap CIs; indirect effects were considered significant when 95% CIs excluded zero.

### 2.7 Statistical Analysis

All analyses used IBM SPSS 22.0 (IBM Corp 2013) and R 4.3.2 (R Core Team 2023). We applied listwise deletion due to low missingness. Given non‐normal distributions (see Table S2), variables were Gaussianized via a nonparanormal transformation using the huge package before computing the zero‐order correlation matrix (Liu et al. 2009; Zhao et al. 2012). The matrix is provided in Table S3.

## 3 Results

### 3.1 Sample Characteristics

Table 1 summarizes the sample characteristics of the final analytic sample (*N =* 6814; 55.09% female; *M*_age_ = 21.08 years, SD_age_ = 3.40). Based on ERratio values, the main sample was divided into two groups: ERratio > 1 (*n =* 3062) and ERratio ≤ 1 (*n =* 3752). Compared with participants in the ERratio ≤ 1 group, participants in the ERratio > 1 group were younger and had fewer years of education (*p'*s < 0.001), showed higher MSS‐Positive, MSS‐Negative, and MSS‐Disorganized trait scores (*p'*s < 0.001), and lower TEPS‐Anticipatory, TEPS‐Consummatory, and MAP scores (*p'*s < 0.001). BAS‐Reward Responsiveness did not differ between groups (*p =* 0.276), whereas BAS‐Drive was slightly higher in ERratio > 1 (*p <* 0.001) and BAS‐Fun seeking was slightly higher in ERratio ≤ 1 (*p =* 0.002).

**TABLE 1:** Descriptive statistics for the present sample.

Variable	ERratio ≤ 1 (*n =* 3752)	ERratio > 1 (*n =* 3062)	Entire sample (*N =* 6814)	*t* (df)^a^	*p*	Hedges' *g* ^b^	95% CI of Hedges' *g*
Age in years	21.44 (3.78)	20.64 (2.80)	21.08 (3.40)	10.59 (6768.2)	< 0.001	0.251	[0.203, 0.299]
Education in years	14.32 (2.16)	13.96 (1.81)	14.16 (2.02)	7.36 (6806.4)	< 0.001	0.176	[0.128, 0.224]
Family history (percentage)							
Afflicted with neurologic or mental disorders	154 (4.10%)	152 (4.97%)	306 (4.50%)	—	—	—	—
None	3525 (93.95%)	2827 (92.42%)	6352 (93.32%)	—	—	—	—
Uncertain	69 (1.84%)	80 (2.62%)	149 (2.19%)	—	—	—	—
Physical disease							
Experienced a major physical disease	28 (0.75%)	21 (0.69%)	49 (0.72%)	—	—	—	—
None	3696 (98.51%)	3015 (98.56%)	6711 (98.59%)	—	—	—	—
Experiencing a major physical disease	10 (0.27%)	10 (0.33%)	20 (0.29%)	—	—	—	—
Others	14 (0.37%)	13 (0.43%)	27 (0.40%)	—	—	—	—
Family income in Chinese yuan (percentage)							
Less than ¥2500	139 (3.70%)	143 (4.67%)	282 (4.14%)	—	—	—	—
¥2501–5000	615 (16.39%)	618 (20.20%)	1233 (18.11%)	—	—	—	—
¥5001–10,000	1279 (34.09%)	1205 (39.39%)	2484 (36.49%)	—	—	—	—
¥10,001–20,000	1227 (32.70%)	838 (27.39%)	2065 (30.34%)	—	—	—	—
¥20,001 and above	488 (13.01%)	255 (8.34%)	743 (10.92%)	—	—	—	—
MAP‐SR	57.17 (9.99)	52.31 (9.66)	54.98 (10.14)	20.34 (6621.0)	< 0.001	0.494	[0.445, 0.542]
TEPS							
Anticipatory subscale	40.63 (6.54)	39.09 (6.62)	39.94 (6.62)	9.55 (6512.0)	< 0.001	0.233	[0.185, 0.281]
Consummatory subscale	48.16 (7.15)	46.96 (7.13)	47.62 (7.17)	6.94 (6550.7)	< 0.001	0.169	[0.121, 0.217]
ERI							
Effort subscale	14.49 (2.87)	19.36 (2.21)	16.68 (3.55)	−79.10 (6789.8)	< 0.001	−1.877	[−1.934, −1.820]
Reward subscale	41.27 (5.09)	35.21 (4.39)	38.54 (5.66)	52.80 (6791.5)	< 0.001	1.267	[1.215, 1.319]
Overcommitment subscale	8.09 (2.44)	8.58 (2.46)	8.31 (2.46)	−8.35 (6514.6)	< 0.001	−0.204	[−0.251, −0.156]
MSS							
Positive schizotypal traits	3.68 (4.64)	6.21 (6.05)	4.81 (5.46)	−19.00 (5647.7)	< 0.001	−0.475	[−0.523, −0.427]
Negative schizotypal traits	5.99 (4.46)	7.81 (4.97)	6.81 (4.78)	−15.79 (6211.4)	< 0.001	−0.389	[−0.437, −0.341]
Disorganized schizotypal traits	3.24 (4.53)	6.22 (6.10)	4.58 (5.49)	−22.46 (5519.8)	< 0.001	−0.563	[−0.612, −0.515]
BAS							
Reward responsiveness	7.05 (2.37)	6.99 (2.14)	7.02 (2.27)	1.09 (6743.6)	0.276	0.026	[−0.021, 0.074]
Drive	7.95 (2.32)	8.18 (2.13)	8.05 (2.24)	−4.27 (6719.6)	< 0.001	−0.103	[−0.151, −0.055]
Fun seeking	10.16 (2.56)	9.97 (2.46)	10.07 (2.52)	3.18 (6634.6)	0.002	0.077	[0.029, 0.125]

*Note:* Continuous variables are presented as mean (SD), and categorical variables as *n* (%).

Abbreviations: BAS, the BAS subscales of the BIS/BAS scale, includes the Fun seeking, Reward Responsiveness, and Drive factors; ERI, the effort–reward imbalance Scale, includes the Effort, Reward, and Overcommitment factors; MAP‐SR, the Motivation and Pleasure Scale‐Self Report, includes the Social, Recreation, Relationship, Motivation factors; MSS, the Multidimensional Schizotypy Scale, includes the positive schizotypal traits, negative schizotypal traits, and disorganized schizotypal traits; TEPS, the Temporal Experience of Pleasure Scale, includes the Anticipatory and Consummatory factors.

^a^ Group comparisons for continuous variables were performed using Welch's *t*‐tests (two‐tailed).

^b^ Effect sizes are Hedges' *g* with 95% confidence intervals; positive values indicate higher scores in the ERratio ≤ 1 group.

### 3.2 Network Structure

**FIGURE 2:**
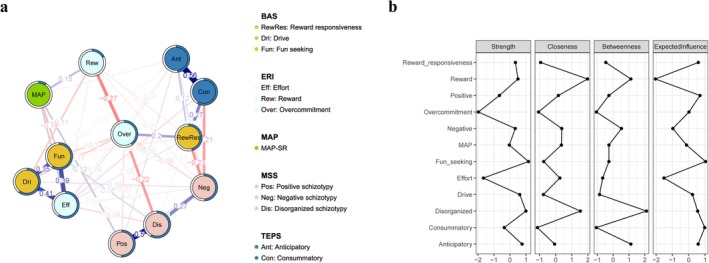
Network structure and centrality estimates of the factors. (a) Network of reward sensitivity, schizotypal‐trait dimensions, the effort–reward imbalance (ERI), reward motivation (MAP‐SR), anticipatory pleasure, and consummatory pleasure at the scale‐factor level (*N =* 6814). (b) Standardized centrality estimates. Note that the nodes represent 12 variables, that is, the factors derived from the BAS, MSS, MAP‐SR, ERI, and TEPS. Edges indicates regularized partial connections between two nodes. Blue and red edges indicate positive and negative connections, respectively. A thicker edge indicates a stronger association between two nodes. The pie chart around a node indicates the predictability of that node in the network. A greater proportion of blue in the pie chart indicates greater predictability of that node. Edge values represent the strength of partial connections.

We investigated the interrelationships among 12 factors (i.e., reward sensitivity, schizotypal trait dimensions, reward motivation, pleasure experience, and ERI) using regularized partial‐correlation networks, as depicted in Figure 2a.

Among the connections between reward sensitivity and schizotypal traits, BAS‐Fun seeking showed a positive connection with MSS‐Disorganized schizotypal traits (edge value = 0.082) and a negative connection with MSS‐Positive schizotypal traits (edge value = −0.031). BAS‐Drive showed positive connections with MSS‐Negative schizotypal traits (edge value = 0.062) and MSS‐Disorganized schizotypal traits (edge value = 0.025). BAS‐Reward Responsiveness showed negative connections with MSS‐Negative schizotypal traits (edge value = −0.232). With respect to motivation, MAP was negatively connected to BAS‐Fun seeking (edge value = −0.141), whereas it exhibited positive connections with MSS‐Positive schizotypal traits (edge value = 0.123), TEPS‐Anticipatory (edge value = 0.036), ERI‐Effort (edge value = 0.111), and ERI‐Reward (edge value = 0.150).

Regarding pleasure experience, TEPS‐Anticipatory displayed positive connections with BAS‐Reward Responsiveness (edge value = 0.118), MSS‐Positive schizotypal traits (edge value = 0.060), MSS‐Negative schizotypal traits (edge value = 0.070), ERI‐Overcommitment (edge value = 0.041), and MAP (edge value = 0.036), but negative connections with BAS‐Drive (edge value = −0.031) and ERI‐Effort (edge value = −0.036). In contrast, TEPS‐Consummatory showed negative connections with MSS‐Negative schizotypal traits (edge value = −0.213) and ERI‐Effort (edge value = −0.077), while exhibiting positive connections with MSS‐Disorganized schizotypal traits (edge value = 0.030) and BAS‐Reward Responsiveness (edge value = 0.268).

### 3.3 Network Centrality Indices

We estimated centrality indices to characterize node prominence within the network (Figure 2b). Notably, BAS‐Fun seeking exhibited the highest strength centrality (*z* = 1.157) and EI (*z* = 1.033), whereas MSS‐Disorganized schizotypal traits showed the highest betweenness centrality (*z* = 2.104). With respect to closeness centrality, the two most central nodes in the network were ERI‐Reward (*z =* 1.974) and MSS‐Disorganized schizotypal traits (*z =* 1.531).

Regarding predictability estimates for individual nodes in the network, BAS‐Reward Responsiveness (0.602), BAS‐Fun seeking (0.590), and BAS‐Drive (0.587) had the highest values. Mean predictability across all nodes was 46.13%, indicating that, on average, 46.13% of each node's variance was explained by its neighboring nodes. Details of all nodes' centrality indices, EI, and predictability in the whole network can be found in Table S1, and the corresponding subgroup‐specific indices can be found in Tables S4 and S5.

### 3.4 Network Stability and Accuracy

We then assessed the stability and accuracy of the network's centrality indices and edge weights (Figures S1 and S2). The correlation stability (CS) coefficient was used to evaluate centrality stability. Notably, all centrality indices as well as edge weights demonstrated high stability: strength (CS coefficient = 0.750), EI (CS coefficient = 0.750), closeness (CS coefficient = 0.750), betweenness (CS coefficient = 0.672), and edge weight (CS coefficient = 0.750). Each of these values substantially exceeded the commonly recommended benchmark of 0.50 for interpretability (Epskamp et al. 2018), indicating stable and interpretable network estimates. The results of the bootstrapped difference tests for node centrality and edge weights are shown in Figures S3–S7.

### 3.5 Mediation Model

Spearman's correlation revealed that BAS‐Fun seeking and MSS‐Disorganized schizotypal traits were significantly positively correlated (*ρ =* 0.054, *p <* 0.001). In contrast, TEPS was significantly negatively correlated with both BAS‐Fun seeking (*ρ =* −0.299, *p < 0*.001) and MSS‐Disorganized schizotypal traits (*ρ =* −0.165, *p <* 0.001). Likewise, MAP was also negatively correlated with BAS‐Fun seeking (*ρ =* −0.158, *p <* 0.001) and MSS‐Disorganized schizotypal traits (*ρ =* −0.333, *p <* 0.001).

**FIGURE 3:**
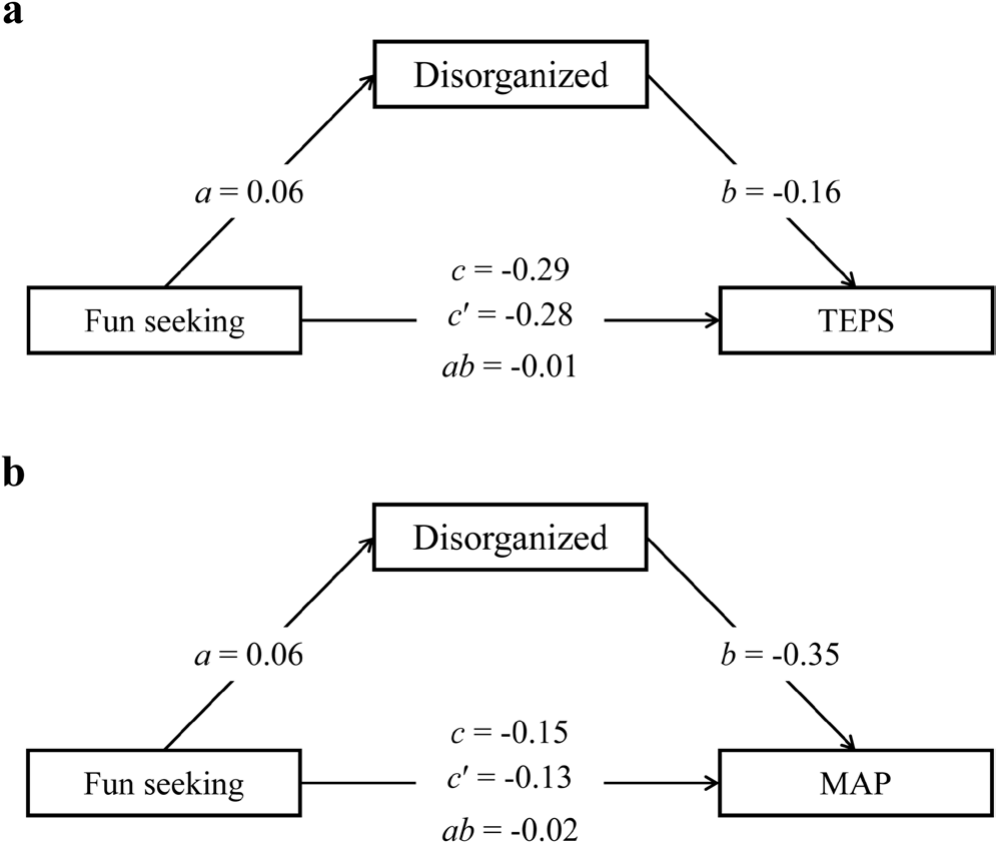
Mediation models among BAS‐Fun seeking, MSS‐disorganized schizotypal traits, pleasure experience (TEPS) and reward motivation (MAP) as unitary constructs. (a) Mediation model with Fun seeking as the independent variable, disorganized schizotypal traits as the mediating variable and TEPS as the dependent variable. (b) Mediation model with Fun seeking as the independent variable, disorganized schizotypal traits as the mediating variable and MAP as the dependent variable. Note that total effects (*c*) and direct effects (*c*′) are also illustrated in the model.

Mediation analysis was conducted (Figure 3) to explore the mediating role of disorganized schizotypal traits in the associations of reward sensitivity with reward motivation and pleasure experience. Regarding TEPS, BAS‐Fun seeking positively predicted MSS‐Disorganized (*a =* 0.0592, 95% bootstrap CI [0.0353, 0.0828], *p <* 0.001). MSS‐Disorganized was negatively associated with TEPS after controlling for BAS‐Fun seeking (*b =* −0.1645, 95% bootstrap CI [−0.1893, −0.1400], *p <* 0.001). The total effect of BAS‐Fun seeking on TEPS was significant (*c =* −0.2860, 95% bootstrap CI [−0.3132, −0.2596], *p <* 0.001), and the direct effect remained significant after accounting for MSS‐Disorganized schizotypal traits (*c*′ *=* −0.2762, 95% bootstrap CI [−0.3031, −0.2496], *p <* 0.001). The indirect effect via MSS‐Disorganized was significant (*ab =* −0.0097, 95% bootstrap CI [−0.0142, −0.0057], *p <* 0.001). For MAP, BAS‐Fun seeking positively predicted MSS‐Disorganized (*a =* 0.0592, 95% bootstrap CI [0.0353, 0.0828], *p <* 0.001). MSS‐Disorganized was negatively associated with MAP after controlling for BAS‐Fun seeking (*b =* −0.3532, 95% bootstrap CI [−0.3768, −0.3294], *p <* 0.001). The total effect of BAS‐Fun seeking on MAP was significant (*c =* −0.1474, 95% bootstrap CI [−0.1740, −0.1209], *p <* 0.001), and the direct effect remained significant after accounting for MSS‐Disorganized (*c*′ *=* −0.1265, 95% bootstrap CI [−0.1521, −0.1014], *p <* 0.001). The indirect effect via MSS‐Disorganized was significant (*ab =* −0.0209, 95% bootstrap CI [−0.0296, −0.0125], *p <* 0.001).

### 3.6 Network Comparison by ERratio Group

**FIGURE 4:**
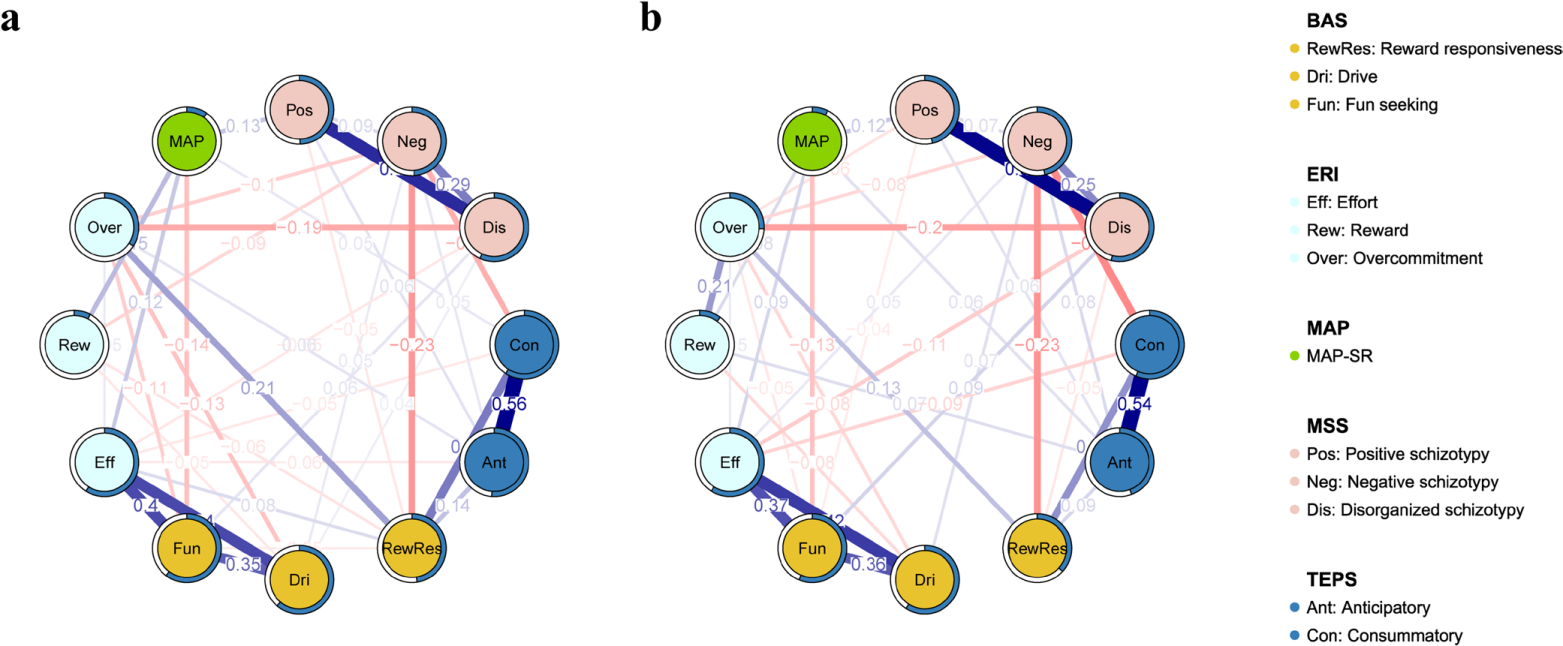
Regularized partial correlation networks for the ERratio ≤ 1 and ERratio > 1 subgroups. (a) Estimated regularized partial network for the ERratio ≤ 1 (*n =* 3752) subgroup. (b) Estimated regularized partial network for the ERratio > 1 (*n =* 3062) subgroup. Note that the nodes represent 12 variables, that is, the factors derived from the BAS, MSS, MAP‐SR, ERI, and TEPS. Edges indicate regularized partial connections between two nodes. Blue and red edges indicate positive and negative connections, respectively. A thicker edge indicates a stronger association between two nodes. The pie chart around a node indicates the predictability of that node in the network. A greater proportion of blue in the pie chart indicates greater predictability of that node. Edge values represent the strength of partial connections.

To examine whether perceived psychosocial stress was associated with differences in the network, we constructed separate networks for subsamples with ERratio ≤ 1 (*n =* 3752) and ERratio > 1 (*n =* 3062). The regularized network structures for both groups are presented in Figure 4. The NCT detected no significant difference in global strength (*S* = 0.309, *p >* 0.05), indicating comparable overall connectivity between the ERratio ≤ 1 and ERratio > 1 networks. In contrast, a significant difference in overall network structure was observed (*M* = 0.197, *p <* 0.001).

At the edge level, several connections showed significant between‐group differences after Holm correction (adjusted *p <* 0.05), including the edge between MSS‐Positive schizotypal traits and MSS‐Disorganized schizotypal traits (*E* = 0.120), the edge between MSS‐Negative schizotypal traits and ERI‐Reward (*E* = 0.100), the edge between TEPS‐Anticipatory and ERI‐Reward (*E* = 0.076), the edge between BAS‐Reward Responsiveness and ERI‐Reward (*E* = 0.053), the edge between BAS‐Fun seeking and ERI‐Overcommitment (*E* = 0.110), and the edge between ERI‐Reward and ERI‐Overcommitment (*E* = 0.197). All remaining edges were not significant after Holm correction (adjusted *p >* 0.05).

Moreover, the ERratio ≤ 1 network demonstrated excellent stability for closeness centrality (CS coefficient = 0.750), edge‐weight (CS coefficient = 0.750), EI (CS coefficient = 0.750), and strength (CS coefficient = 0.750), and showed good stability for betweenness centrality (CS coefficient = 0.517). In contrast, the ERratio > 1 network showed good stability for betweenness (CS coefficient = 0.517) and closeness centrality (CS coefficient = 0.517) and exhibited excellent stability for edge‐weight (CS coefficient = 0.750), EI (CS coefficient = 0.750), and strength (CS coefficient = 0.750). Additional network comparison metrics are presented in Figures S8 and S9.

## 4 Discussion

This study applied network analysis to examine the multivariate associations among reward processing, schizotypal traits, and psychosocial stress in a large community sample. Three key findings emerged: (1) BAS‐Fun seeking exhibited the highest strength centrality and EI; (2) MSS‐Disorganized schizotypal traits served as a bridge connecting reward sensitivity to motivation and pleasure, and supplementary mediation analyses showed negative indirect effects; and (3) psychosocial stress (ERI) was associated with significant differences in network structure but not in global connectivity strength.

BAS‐Fun seeking ranked highest in both strength and EI. Compared with Drive and Reward Responsiveness, Fun seeking reflects a composite tendency centered on novelty seeking, immediate approach, and impulsivity (Carver and White 1994; Smillie et al. 2006). It aligns more closely with cue‐elicited approach impulses and “wanting” rather than with sustained goal persistence (Carver and White 1994; Smillie et al. 2006; Berridge and Robinson 1998). This may make it more likely to exhibit broad associations with reward motivation and multiple trait dimensions (Smillie et al. 2006; Leone and Russo 2009).

Disorganized schizotypal traits demonstrated the highest betweenness and the second‐highest closeness centrality and played a mediating role between BAS‐Fun seeking and both MAP and TEPS. Specifically, when Fun seeking is high and accompanied by higher disorganized traits, MAP and TEPS scores tend to be lower. One plausible explanation is a construct‐level dissociation. Fun seeking emphasizes impulsive novelty pursuit, whereas MAP and TEPS focus more on sustained, goal‐directed motivation and trait pleasure capacity (Llerena et al. 2013; Wang et al. 2020; Gard et al. 2006; Chan et al. 2012). When cognitive and behavioral organization is limited, a stronger impulsive approach tendency may not necessarily translate into higher levels of sustained motivation and pleasure capacity (Hofmann et al. 2012; Diamond 2013; Duckworth and Gross 2014; Steffens et al. 2018; Karamaouna et al. 2024). The Strength Model of Self‐Control offers a potential framework, positing that insufficient self‐regulatory resources constrain the transformation of impulsive approach into sustained, effortful goal pursuit (Baumeister et al. 2007, 1998).

The NCT indicated no significant difference in global strength between the high‐ and low‐stress groups, but it did show a significant difference in overall network structure. Several specific edge weights remained significantly different after Holm correction. Stress‐related inflammatory and neuroendocrine activity can modulate reward processing, including reward sensitivity, motivation, and pleasure experience, in a component‐specific manner (Baik 2020; Boyle et al. 2023). Such component‐specific perturbations can selectively reweight multivariable associations without necessarily changing overall connectedness (Snippe et al. 2017). Stress responsivity varies across individuals and task contexts, with evidence for both reduced reward sensitivity and enhanced reward learning under stress (Berghorst et al. 2013; Lighthall et al. 2013).

Given that the sample primarily comprised young adults, the network association structure may be developmentally specific. During late adolescence and young adulthood, responsiveness to reward cues and risk preference often increase, whereas prefrontal cognitive control undergoes continued maturation (Spear 2000; Casey et al. 2008; Galván 2010; Steinberg 2010; Somerville and Casey 2010). The stress‐response system is likewise undergoing sensitive changes in young adults, particularly in HPA‐axis reactivity (Gunnar and Quevedo 2007; Romeo 2013), and stress effects vary by developmental timing (Lupien et al. 2009); stress can also modulate dopaminergic reward circuitry (Baik 2020). In addition, psychosis‐like experiences are more prevalent in young adulthood within the general population (Kelleher et al. 2012; Yates et al. 2021; Staines et al. 2022), and developmental stage may therefore influence how psychosis‐proneness relates to reward processing (van Os et al. 2009; Papanastasiou et al. 2018; Harju‐Seppänen et al. 2022).

Several limitations should be noted. The cross‐sectional design precludes causal inference, and cross‐sectional networks may mix stable between‐person differences with within‐person fluctuations, limiting conclusions about stress‐related coupling over time; longitudinal data are needed. Because participants in the present sample were mostly young adults, generalization beyond this age range should be made cautiously. Future studies should employ an independent validation sample with a wider age range to test the robustness of the network against variations in sampling sources, measurement choices, and node definitions. All variables were self‐reported, which may introduce recall bias and common‐method variance; future studies should incorporate behavioral tasks and interview‐ or clinician‐rated assessments.

Overall, this study provides a multivariate conditional association map linking reward sensitivity, schizotypal traits, reward motivation, and pleasure experience in a large nonclinical sample. BAS‐Fun seeking showed the highest strength centrality and EI, and MSS‐Disorganized traits showed a bridging role, suggesting that disorganization may shape how cue‐driven approach tendencies relate to sustained motivation and pleasure. ERI‐based comparisons suggest that psychosocial stress may relate to selective changes in specific associations rather than to a uniform change in overall connectedness. Future work should use longitudinal and multi‐method designs, model ERI continuously, and integrate behavioral and neurobiological measures to strengthen mechanistic inference and generalizability.

## 5 Conclusion

This study used network analysis to characterize multivariate associations among reward sensitivity (BAS), schizotypal traits (MSS), reward motivation (MAP‐SR), pleasure experience (TEPS), and psychosocial stress (ERI) in a large Chinese young adult sample. BAS‐Fun seeking was the most central node, and MSS‐Disorganized traits bridged BAS‐Fun seeking with TEPS and MAP‐SR, with supplementary mediation analyses indicating indirect pathways consistent with the network pattern. Networks showed similar overall connectivity but different structures between low‐ and high‐ psychosocial‐stress groups. These findings provide a concise descriptive framework and motivate longitudinal, multi‐method studies to test temporal mechanisms and stress‐related contingencies across the psychosis continuum.

## Funding

This study was supported by the Scientific Foundation of the Institute of Psychology, Chinese Academy of Sciences (E2CX3415CX), the National Science Foundation of China (32471138), and the Philip K.H. Wong Foundation.

## Conflicts of Interest

The authors declare no conflicts of interest.

## Supporting information


**Table S1:** Centrality, predictability, expected influence and predictability of nodes in the whole network (*n* = 6814).
**Table S2:** Normality test of variables in the whole network (*n* = 6814).
**Table S3:** Zero‐order correlation matrix of variables selected for the whole network (*n* = 6814).
**Table S4:** Centrality, predictability, expected influence, and predictability of nodes in the ERratio < 1 network (*n* = 3673).
**Table S5:** Centrality, predictability, expected influence, and predictability of nodes in the ERratio > 1 network (*n* = 3062).
**Figure S1:** Average correlation between centrality indices of the original whole sample and those estimated in subgroups obtained by dropping increasing percentages of subjects for the whole network.
**Figure S2:** Bootstrapped confidence intervals of estimated edge‐weights for the whole network.
**Figure S3:** Bootstrapped difference test for node strength centrality in the whole network.
**Figure S4:** Bootstrapped difference test for node betweenness centrality in the whole network.
**Figure S5:** Bootstrapped difference test for node closeness centrality in the whole network.
**Figure S6:** Bootstrapped difference test for node expected influence centrality in the whole network.
**Figure S7:** Bootstrapped difference tests between edge‐weights in the whole network.
**Figure S8:** Average correlation between centrality indices of the original whole sample and those estimated in subgroups obtained by dropping increasing percentages of subjects for the ERratio > 1 network.
**Figure S9:** Average correlation between centrality indices of the original whole sample and those estimated in subgroups obtained by dropping increasing percentages of subjects for the ERratio < 1 network.

## Data Availability

The data that support the findings of this study are available on request from the corresponding author. The data are not publicly available due to privacy or ethical restrictions.
