# Supplementary material for: Interplay Among Reward Processing, Schizotypal Traits, and Psychosocial Stress in a Large Chinese Young Adult Sample: A Cross‐Sectional Network Analysis
Source: Psych J. 2026 May 20;15(3):e70102. doi: 10.1002/pchj.70102 (PMC13240555; doi:10.1002/pchj.70102)
Supplement: Supplementary file 1 — Table S1: Centrality, predictability, expected influence and predictability of nodes in the whole network (n = 6814). Table S2: Normality test of variables in the whole network (n = 6814). Table S3: Zero‐order correlation matrix of variables selected for the whole network (n = 6814). Table S4: Centrality, predictability, expected influence, and predictability of nodes in the ERratio < 1 network (n = 3673). Table S5: Centrality, predictability, expected influence, and predictability of nodes in the ERratio > 1 network (n = 3062). Figure S1: Average correlation between centrality indices of the original whole sample and those estimated in subgroups obtained by dropping increasing percentages of subjects for the whole network. Figure S2: Bootstrapped confidence intervals of estimated edge‐weights for the whole network. Figure S3: Bootstrapped difference test for node strength centrality in the whole network. Figure S4: Bootstrapped difference test for node betweenness centrality in the whole network. Figure S5: Bootstrapped difference test for node closeness centrality in the whole network. Figure S6: Bootstrapped difference test for node expected influence centrality in the whole network. Figure S7: Bootstrapped difference tests between edge‐weights in the whole network. Figure S8: Average correlation between centrality indices of the original whole sample and those estimated in subgroups obtained by dropping increasing percentages of subjects for the ERratio > 1 network. Figure S9: Average correlation between centrality indices of the original whole sample and those estimated in subgroups obtained by dropping increasing percentages of subjects for the ERratio < 1 network. [file PCHJ-15-e70102-s001.zip › 0Supplementary File_31_07_ TDH_hj.docx]

Supplementary Materials

Supplementary Methods

Centrality estimates

To evaluate the importance of each node, four centrality indices were employed: strength, betweenness, closeness, and expected influence (Borsboom & Cramer, 2013; Epskamp et al., 2018). strength refers to the sum of absolute weights of a node’s direct connections, indicating its overall connectedness. closeness represents the inverse of the weighted sum of shortest-path lengths between that node and all others, reflecting its average proximity to the network. betweenness measures the number of shortest paths between node pairs that pass through a given node, highlighting its intermediary role. expected influence is determined by summing all of a node’s edge weights, including negative weights (Robinaugh et al., 2016). Additionally, predictability was calculated as the proportion of variance in each node explained by all other nodes in the network (Haslbeck & Waldorp, 2020).

Network stability and accuracy

Following the methodology outlined by Epskamp et al. (2018), the stability of the network was assessed using the case-dropping bootstrap procedure. In this approach, if a substantial proportion of cases can be removed from the dataset without substantially altering any node’s centrality indices, the network is considered stable. Stability was both visually represented and quantified via correlation stability (CS) coefficients. The accuracy of edge weights was evaluated through a nonparametric bootstrap procedure, calculating 95% confidence intervals (CIs). Observations were randomly resampled to generate 5,000 bootstrap samples, from which the CIs were computed. Finally, a bootstrap difference test was applied to assess differences in network properties.

Supplementary Tables

| Supplementary Table S1. Centrality, predictability, expected influence and predictability of nodes in the whole network (n = 6814) | | | | | |
| --- | --- | --- | --- | --- | --- |
|  | Strength | Closeness | Betweenness | Expected  Influence | Predictability |
| BAS – Reward responsiveness | 0.341 | –0.928 | –0.460 | 0.589 | 0.602 |
| BAS – Drive | 0.613 | –0.757 | –0.855 | 0.231 | 0.587 |
| BAS – Fun seeking | 1.157 | –0.738 | –0.263 | 1.033 | 0.590 |
| MAP-SR | –0.040 | 0.358 | –0.263 | –0.135 | 0.457 |
| MSS – Positive schizotypy | –0.659 | 0.173 | –0.263 | 0.693 | 0.502 |
| MSS – Negative schizotypy | 0.329 | 0.379 | 0.526 | –0.975 | 0.490 |
| MSS – Disorganized schizotypy | 0.998 | 1.532 | 2.104 | 0.549 | 0.577 |
| ERI – Effort | –1.661 | 0.263 | –0.658 | –1.519 | 0.174 |
| ERI – Reward | 0.501 | 1.974 | 1.118 | –2.043 | 0.399 |
| ERI – Overcommitment | –1.978 | –1.062 | –1.052 | 0.015 | 0.094 |
| TEPS – Anticipatory | 0.759 | –0.063 | 1.118 | 0.578 | 0.582 |
| TEPS – Consummatory | –0.360 | –1.130 | –1.052 | 0.983 | 0.481 |

Note: BAS: the BAS subscale; MAP-SR: the Motivation and Pleasure Scale - Self-Report; MSS: the Multidimensional Schizotypy Scale; ERI: the Effort - Reward Imbalance Scale; TEPS: the Temporal Experience of Pleasure Scale.

| Supplementary Table S2. Normality test of variables in the whole network (n = 6814) | | |
| --- | --- | --- |
| Nodes | Kolmogorov – Smirnov normality test | |
|  | D | *p* |
| BAS – Reward responsiveness | 0.153 | 3.71×10^-140^ |
| BAS – Drive | 0.123 | 1.55×10^-90^ |
| BAS – Fun seeking | 0.112 | 9.16×10^-75^ |
| MAP-SR | 0.040 | 9.01×10^-10^ |
| MSS – Positive schizotypy | 0.189 | 5.25×10^-214^ |
| MSS – Negative schizotypy | 0.118 | 9.07×10^-84^ |
| MSS – Disorganized schizotypy | 0.218 | 4.55×10^-284^ |
| ERI – Effort | 0.081 | 6.31×10^-39^ |
| ERI – Reward | 0.056 | 2.64×10^-19^ |
| ERI – Overcommitment | 0.102 | 2.40×10^-62^ |
| TEPS – Anticipatory | 0.051 | 5.82×10^-16^ |
| TEPS – Consummatory | 0.054 | 1.16×10^-17^ |
| Note: *p* < 0.001 are bold. Two - tailed One - Sample Kolmogorov - smirnov test with the Lilliefors significance correction was conducted. | | |

| Supplementary Table S3. Zero - order correlation matrix of variables selected for the whole network (n = 6814) | | | | | | | | | | | | |  |
| --- | --- | --- | --- | --- | --- | --- | --- | --- | --- | --- | --- | --- | --- |
|  | | MSS –  Positive schizotypy | MSS –  Negative schizotypy | MSS –  Disorganized schizotypy | TEPS –  Consummatory | TEPS –  Anticipatory | BAS –  Reward responsiveness | BAS –  Drive | BAS –  Fun seeking | ERI –  Effort | ERI –  Reward | ERI –  Overcommitment | MAP |
| MSS –  Positive schizotypy | 1 | – | – | – | – | – | – | – | – | – | – | – |  |
| MSS –  Negative schizotypy | 0.376 | 1 | – | – | – | – | – | – | – | – | – | – |  |
| MSS –  Disorganized schizotypy | 0.636 | 0.516 | 1 | – | – | – | – | – | – | – | – | – |  |
| TEPS –  Consummatory | -0.082 | -0.439 | -0.187 | 1 | – | – | – | – | – | – | – | – |  |
| TEPS –  Anticipatory | -0.009 | -0.265 | -0.127 | 0.682 | 1 | – | – | – | – | – | – | – |  |
| BAS –  Reward responsiveness | -0.230 | -0.506 | -0.340 | 0.554 | 0.445 | 1 | – | – | – | – | – | – |  |
| BAS –  Drive | 0.090 | 0.274 | 0.189 | -0.250 | -0.251 | -0.216 | 1 | – | – | – | – | – |  |
| BAS –  Fun seeking | 0.079 | 0.239 | 0.204 | -0.272 | -0.257 | -0.248 | 0.673 | 1 | – | – | – | – |  |
| ERI –  Effort | 0.016 | 0.204 | 0.062 | -0.268 | -0.250 | -0.145 | 0.679 | 0.661 | 1 | – | – | – |  |
| ERI –  Reward | 0.152 | 0.066 | 0.169 | -0.027 | 0.001 | -0.138 | -0.126 | -0.069 | -0.128 | 1 | – | – |  |
| ERI –  Overcommitment | -0.368 | -0.427 | -0.494 | 0.273 | 0.236 | 0.445 | -0.267 | -0.284 | -0.136 | -0.298 | 1 | – |  |
| MAP | 0.155 | -0.002 | 0.058 | 0.100 | 0.109 | 0.066 | -0.036 | -0.123 | 0.002 | 0.167 | -0.016 | 1 |  |
| Note: BAS: the BAS subscale; MAP - SR: the Motivation and Pleasure Scale - Self-Report; MSS: the Multidimensional Schizotypy Scale; ERI: the Effort - Reward Imbalance Scale; TEPS: the Temporal Experience of Pleasure Scale. | | | | | | | | | | | | |  |

| Supplementary Table S4. Centrality, predictability, expected influence and predictability of nodes in the ERratio < 1 network (n = 3673) | | | | | |
| --- | --- | --- | --- | --- | --- |
|  | Strength | Closeness | Betweenness | Expected  Influence | Predictability |
| BAS – Reward responsiveness | 0.252 | -0.622 | -0.761 | 0.640 | 0.608 |
| BAS – Drive | 0.633 | -0.571 | -0.761 | 0.241 | 0.601 |
| BAS – Fun seeking | 1.082 | -0.400 | -0.951 | 1.226 | 0.590 |
| MAP-SR | 0.683 | 1.198 | 0.190 | -0.225 | 0.472 |
| MSS – Positive schizotypy | -0.341 | 0.442 | 0 | 0.596 | 0.492 |
| MSS – Negative schizotypy | 0.520 | 0.926 | 1.522 | -1.404 | 0.487 |
| MSS – Disorganized schizotypy | 0.539 | 1.238 | 1.712 | 0.501 | 0.564 |
| ERI – Effort | -2.374 | -1.859 | -1.141 | -1.250 | 0.077 |
| ERI – Reward | -0.242 | 1.178 | 0.951 | -1.801 | 0.341 |
| ERI – Overcommitment | -1.413 | -0.582 | 0 | -0.235 | 0.102 |
| TEPS – Anticipatory | 0.726 | 0.015 | 0.380 | 0.657 | 0.585 |
| TEPS – Consummatory | -0.063 | -0.963 | -1.141 | 1.054 | 0.505 |

Notes: BAS: the BAS subscale; MAP-SR: the Motivation and Pleasure Scale - Self-Report; MSS: the Multidimensional Schizotypy Scale; ERI: the Effort - Reward Imbalance Scale; TEPS: the Temporal Experience of Pleasure Scale.

| Supplementary Table S5. Centrality, predictability, expected influence and predictability of nodes in the ERratio > 1 network (n = 3062) | | | | | |
| --- | --- | --- | --- | --- | --- |
|  | Strength | Closeness | Betweenness | Expected  influence | Predictability |
| BAS – Reward responsiveness | 0.338 | -0.604 | -0.210 | 0.782 | 0.592 |
| BAS – Drive | 0.508 | -0.663 | -0.629 | 0.539 | 0.563 |
| BAS – Fun seeking | 0.993 | -0.095 | 0.629 | 1.079 | 0.591 |
| MAP-SR | -0.691 | -0.352 | -0.629 | -0.798 | 0.368 |
| MSS – Positive schizotypy | -0.105 | 0.900 | -0.210 | 0.800 | 0.464 |
| MSS – Negative schizotypy | 0.663 | 0.887 | 0.420 | -1.478 | 0.464 |
| MSS – Disorganized schizotypy | 1.282 | 2.277 | 2.307 | 0.290 | 0.540 |
| ERI – Effort | -1.863 | -1.156 | -1.049 | -0.445 | 0.102 |
| ERI – Reward | -0.165 | 0.114 | -0.210 | -1.599 | 0.257 |
| ERI – Overcommitment | -1.711 | -1.302 | -1.049 | -0.667 | 0.074 |
| TEPS – Anticipatory | 0.802 | 0.342 | 1.259 | 0.018 | 0.566 |
| TEPS – Consummatory | -0.050 | -0.347 | -0.629 | 1.479 | 0.443 |

Notes: BAS: the BAS subscale; MAP-SR: the Motivation and Pleasure Scale - Self-Report; MSS: the Multidimensional Schizotypy Scale; ERI: the Effort - Reward Imbalance Scale; TEPS: the Temporal Experience of Pleasure Scale.

Supplementary Figures

| 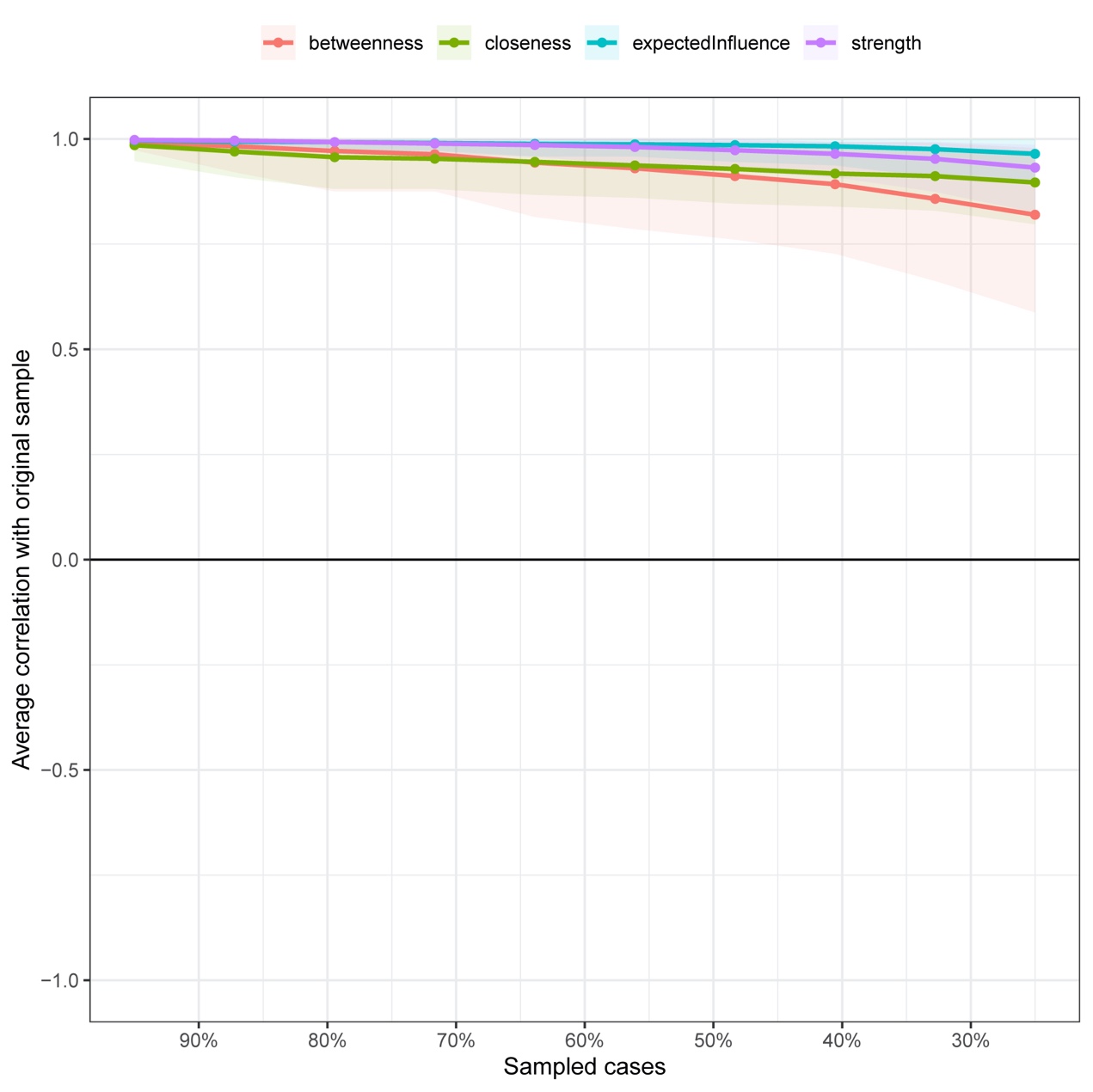 |
| --- |
| Supplementary Figure S1. Average correlation between centrality indices of the original whole sample and those estimated in subgroups obtained by dropping increasing percentages of subjects for the whole network  Note: The lines indicate the means and the areas indicate the range from 2.5% to 97.5% quantile. |

| 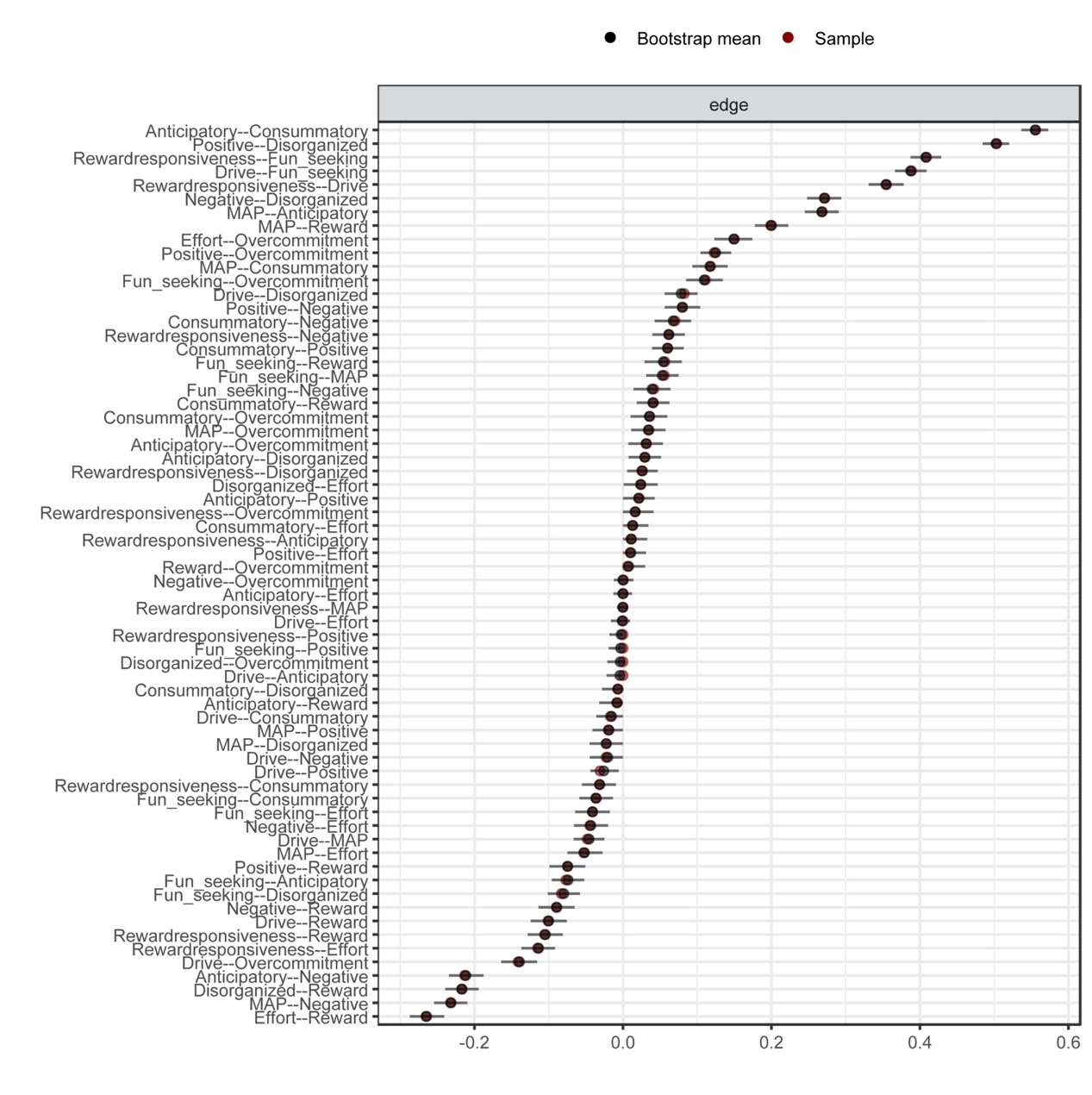 |
| --- |
| Supplementary Figure S2. Bootstrapped confidence intervals of estimated edge-weights for the whole network |

| 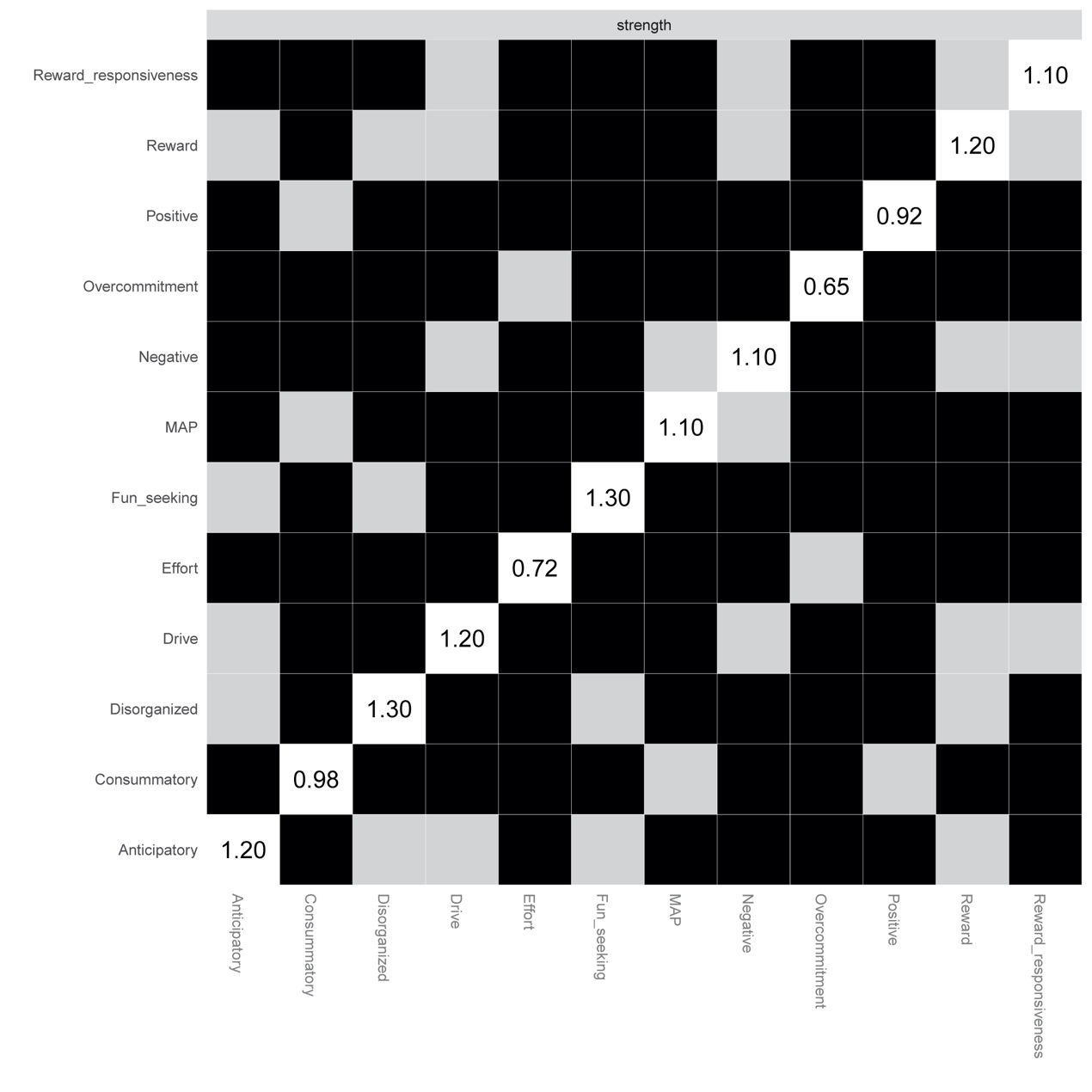 |
| --- |
| Supplementary Figure S3. Bootstrapped difference test for node strength centrality in the whole network  Note: Black boxes indicate strengths that are significantly different from one another, and grey boxes indicate strengths that are not significantly different. |

| 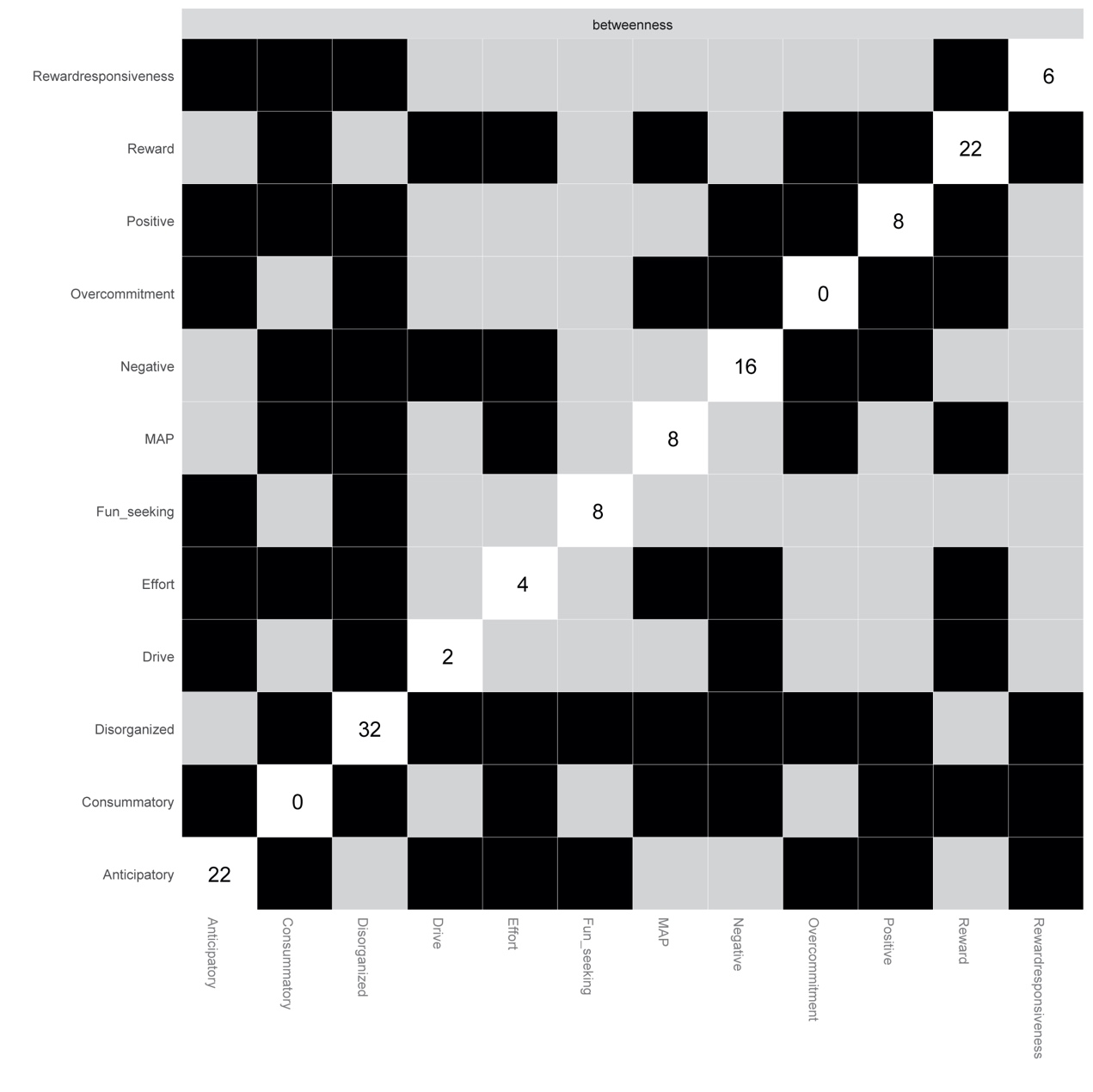 |
| --- |
| Supplementary Figure S4. Bootstrapped difference test for node betweenness centrality in the whole network  Note: Black boxes indicate strengths that are significantly different from one another, and grey boxes indicate strengths that are not significantly different. |

| 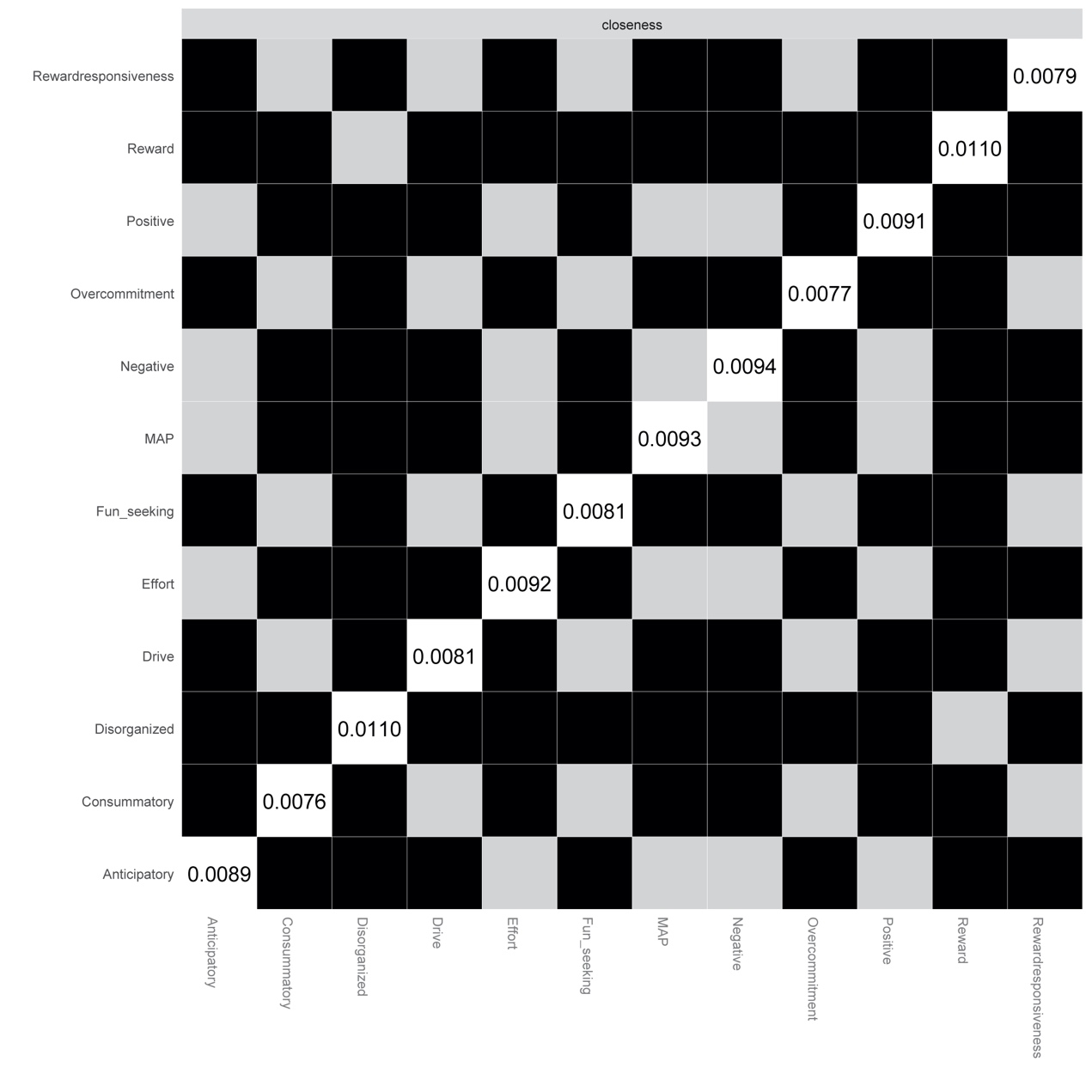 |
| --- |
| Supplementary Figure S5. Bootstrapped difference test for node closeness centrality in the whole network  Note: Black boxes indicate strengths that are significantly different from one another, and grey boxes indicate strengths that are not significantly different. |
| 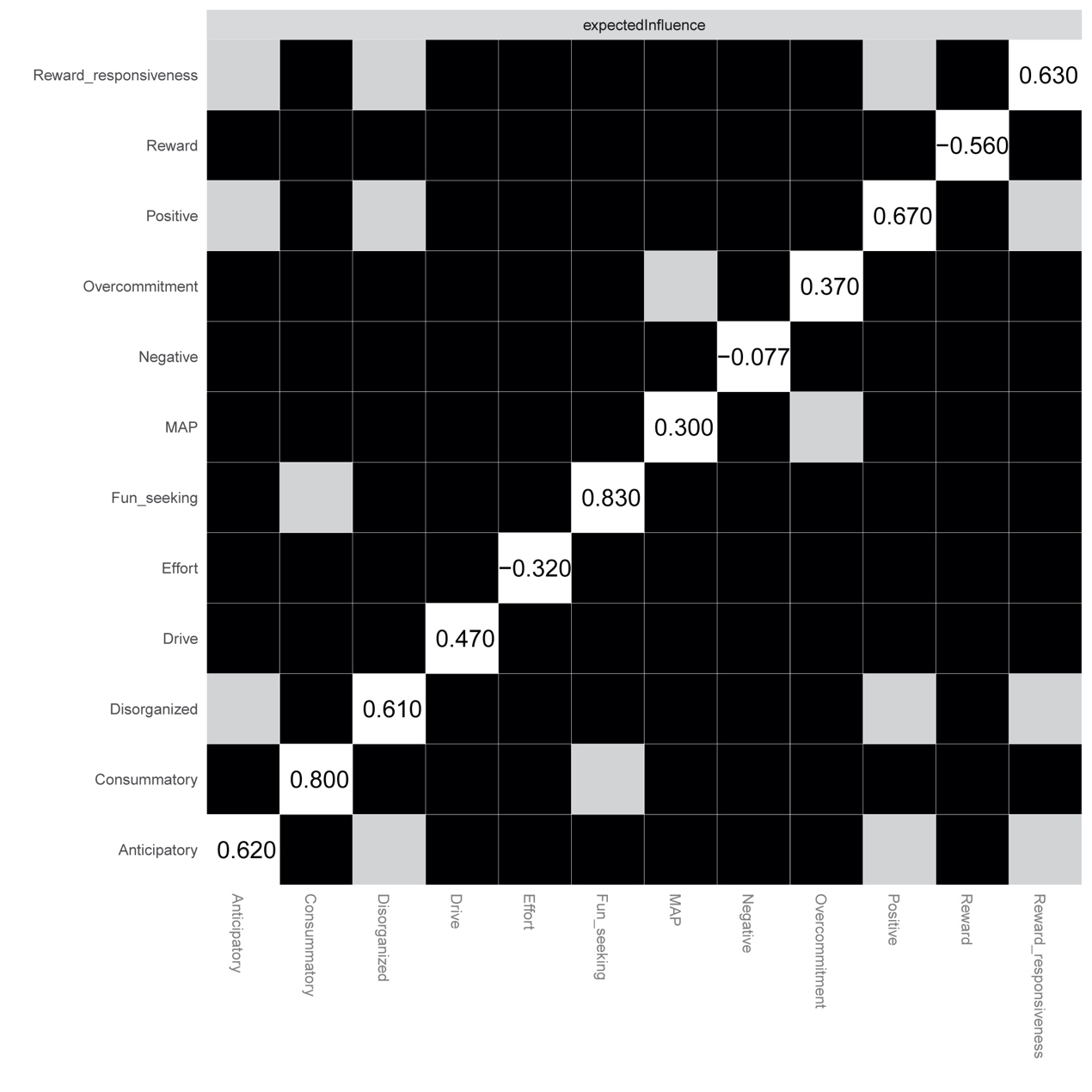 |
| Supplementary Figure S6. Bootstrapped difference test for node expected influence centrality in the whole network  Note: Black boxes indicate expected influence (EI) that are significantly different from one another, and grey boxes indicate EI that are not significantly different. |

| 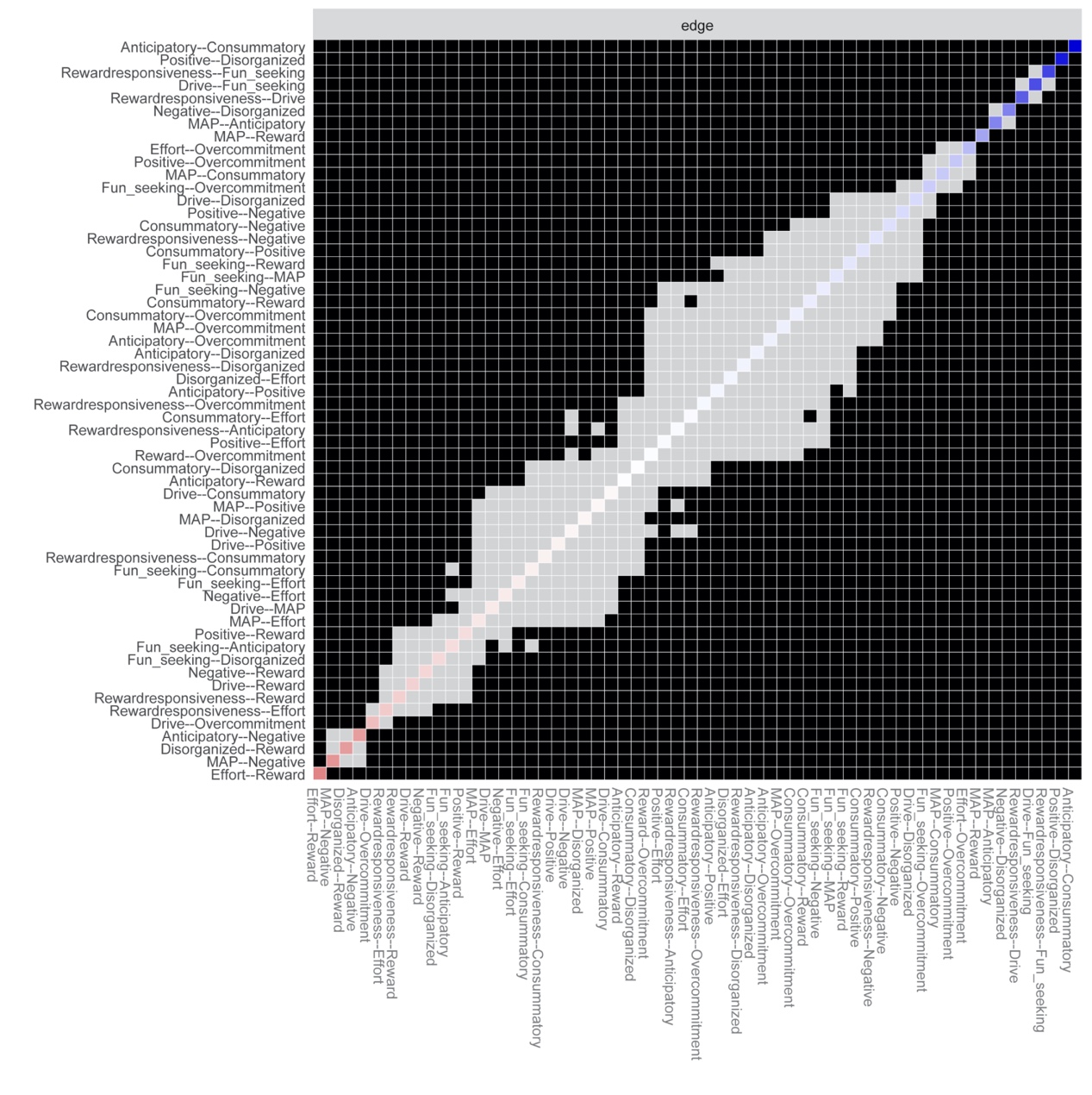 |
| --- |
| Supplementary Figure S7. Bootstrapped difference tests between edge-weights in the whole network  Note: Black boxes indicate expected influence (EI) that are significantly different from one another, and grey boxes indicate EI that are not significantly different. |

| 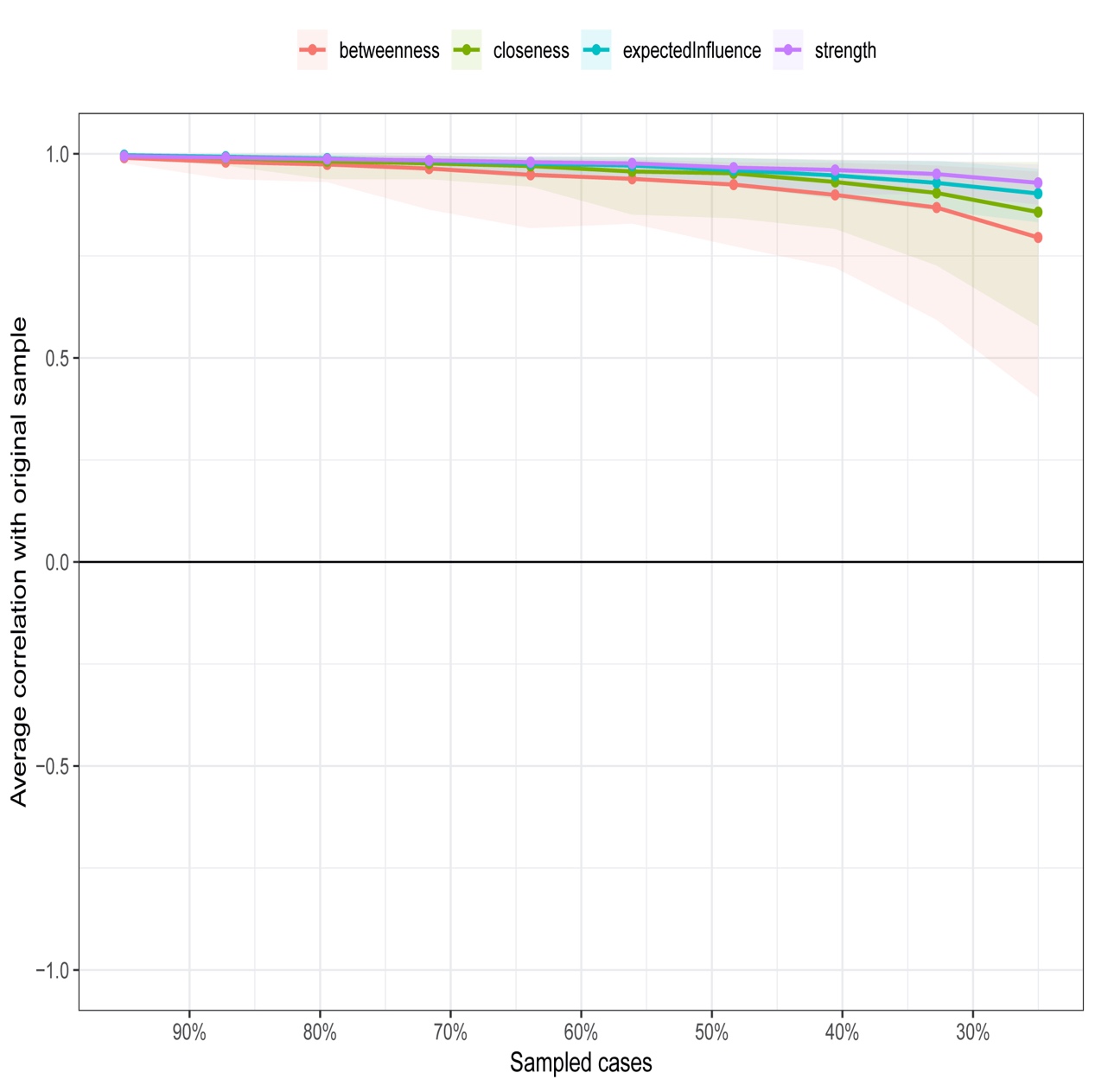 |
| --- |
| Supplementary Figure S8. Average correlation between centrality indices of the original whole sample and those estimated in subgroups obtained by dropping increasing percentages of subjects for the ERratio > 1 network  Note: The lines indicate the means and the areas indicate the range from 2.5% to 97.5% quantile. |

| 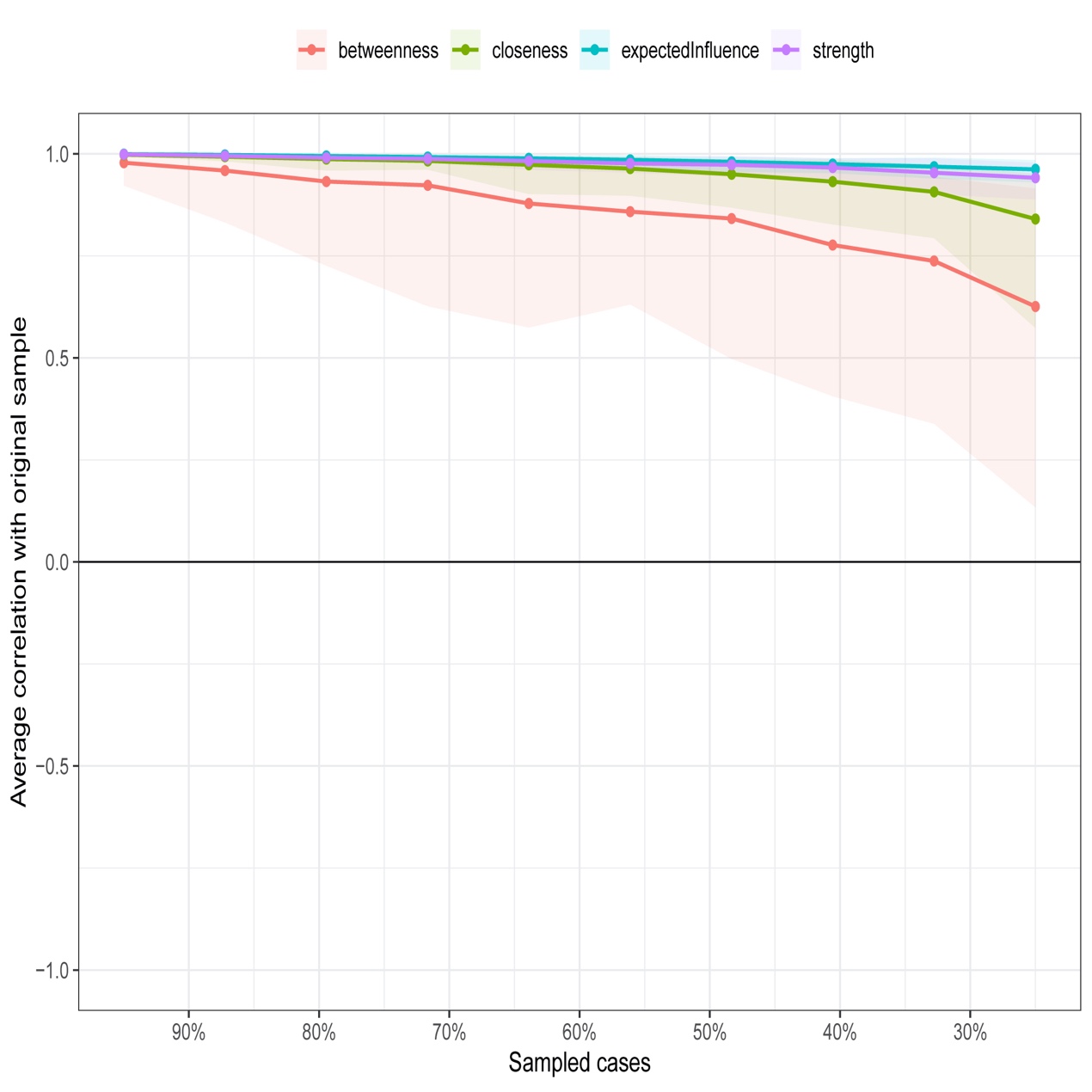 |
| --- |
| Supplementary Figure S9. Average correlation between centrality indices of the original whole sample and those estimated in subgroups obtained by dropping increasing percentages of subjects for the ERratio < 1 network  Note: The lines indicate the means and the areas indicate the range from 2.5% to 97.5% quantile. |

Supplementary References

Borsboom, D. & Cramer, A. O. J. Network analysis: an integrative approach to the structure of psychopathology. Ann. Rev. Clin. psychol. 9, 91 - 121 (2013).

Epskamp, S., Borsboom, D. & Fried, E. I. Estimating psychological networks and their accuracy: A tutorial paper. Behav. Res. Methods 50, 195 - 212 (2018).

Haslbeck, J. & Fried, E. I. How predictable are symptoms in psychopathological networks? A reanalysis of 18 published datasets. Psychol. Med. 47, 2767–2776 (2017).

Haslbeck, J. M. B., & Waldorp, L. J. mgm: Estimating Time - Varying Mixed Graphical Models in High - Dimensional Data. J. Stat. Softw. 93(8), 1–46 (2020).

Robinaugh, D. J., Millner, A. J. & McNally, R. J. Identifying highly influential nodes in the complicated grief network. J. Abnorm. Psychol. 125, 747 - 757 (2016).
