# Supplementary figures and images for: Interplay Among Reward Processing, Schizotypal Traits, and Psychosocial Stress in a Large Chinese Young Adult Sample: A Cross‐Sectional Network Analysis
Source: Psych J. 2026 May 20;15(3):e70102. doi: 10.1002/pchj.70102 (PMC13240555; doi:10.1002/pchj.70102)

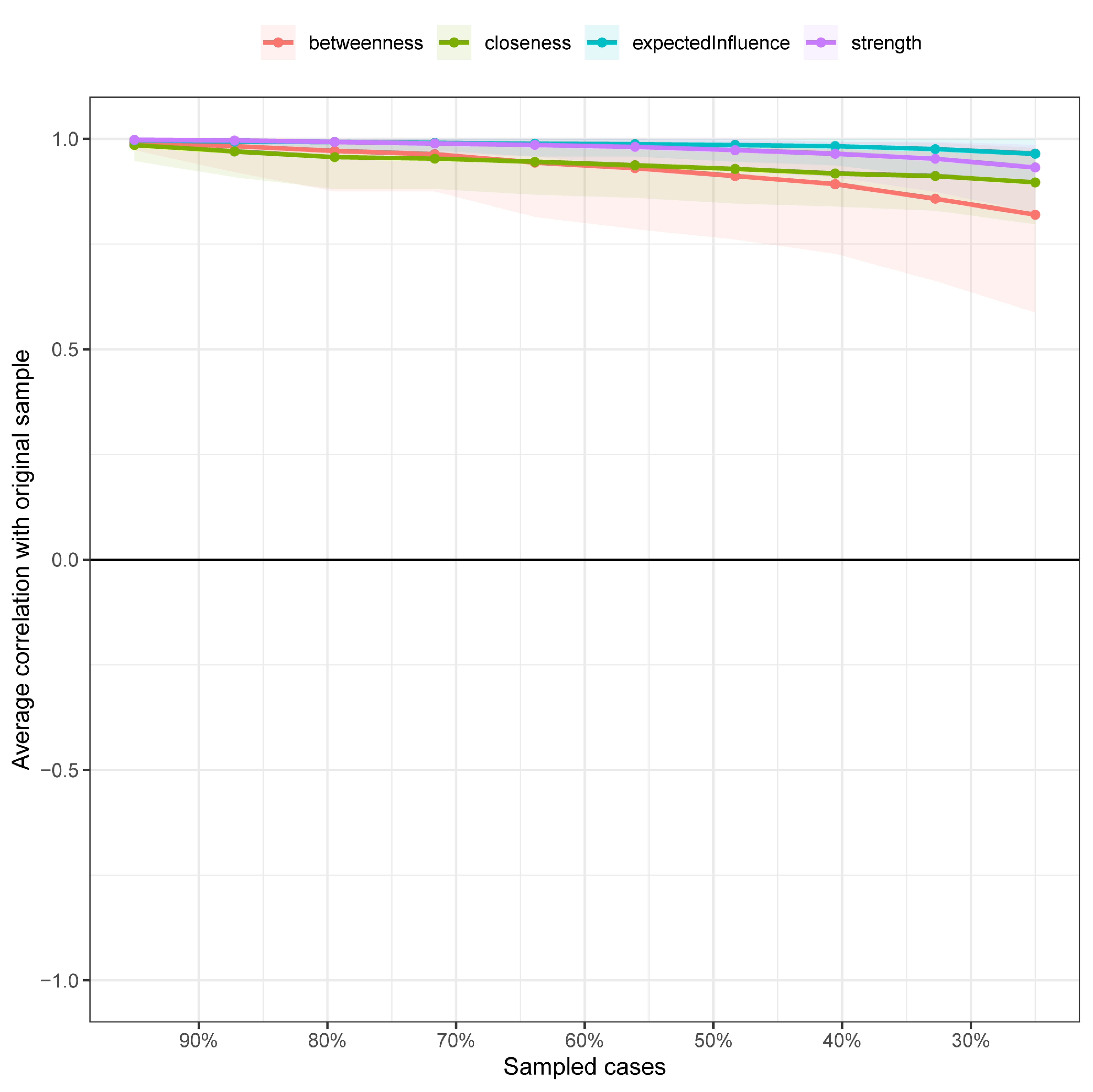

Supplement: Supplementary file 1 — Table S1: Centrality, predictability, expected influence and predictability of nodes in the whole network (n = 6814). Table S2: Normality test of variables in the whole network (n = 6814). Table S3: Zero‐order correlation matrix of variables selected for the whole network (n = 6814). Table S4: Centrality, predictability, expected influence, and predictability of nodes in the ERratio < 1 network (n = 3673). Table S5: Centrality, predictability, expected influence, and predictability of nodes in the ERratio > 1 network (n = 3062). Figure S1: Average correlation between centrality indices of the original whole sample and those estimated in subgroups obtained by dropping increasing percentages of subjects for the whole network. Figure S2: Bootstrapped confidence intervals of estimated edge‐weights for the whole network. Figure S3: Bootstrapped difference test for node strength centrality in the whole network. Figure S4: Bootstrapped difference test for node betweenness centrality in the whole network. Figure S5: Bootstrapped difference test for node closeness centrality in the whole network. Figure S6: Bootstrapped difference test for node expected influence centrality in the whole network. Figure S7: Bootstrapped difference tests between edge‐weights in the whole network. Figure S8: Average correlation between centrality indices of the original whole sample and those estimated in subgroups obtained by dropping increasing percentages of subjects for the ERratio > 1 network. Figure S9: Average correlation between centrality indices of the original whole sample and those estimated in subgroups obtained by dropping increasing percentages of subjects for the ERratio < 1 network. [file PCHJ-15-e70102-s001.zip › supplementary Figure 1.png]

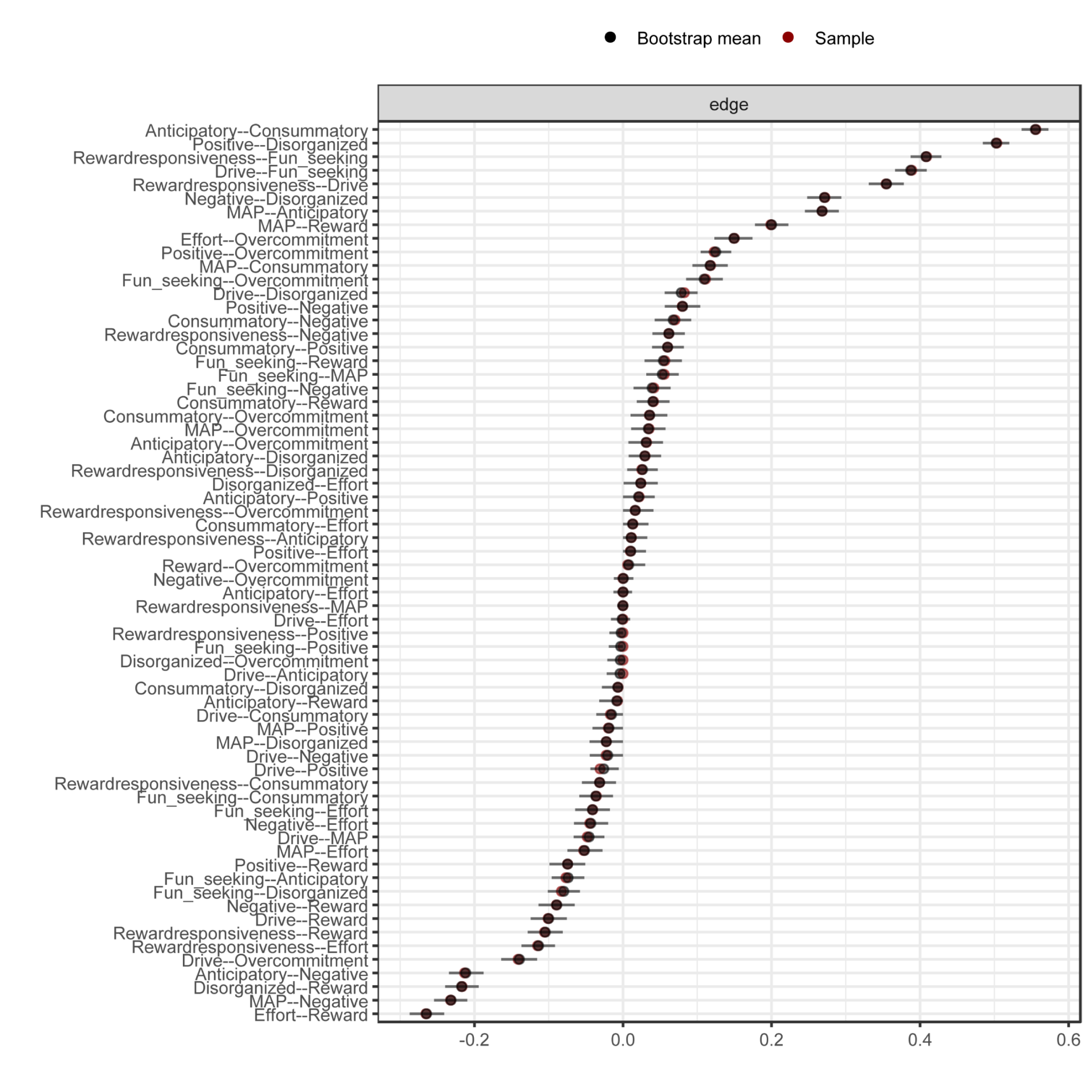

Supplement: Supplementary file 1 — Table S1: Centrality, predictability, expected influence and predictability of nodes in the whole network (n = 6814). Table S2: Normality test of variables in the whole network (n = 6814). Table S3: Zero‐order correlation matrix of variables selected for the whole network (n = 6814). Table S4: Centrality, predictability, expected influence, and predictability of nodes in the ERratio < 1 network (n = 3673). Table S5: Centrality, predictability, expected influence, and predictability of nodes in the ERratio > 1 network (n = 3062). Figure S1: Average correlation between centrality indices of the original whole sample and those estimated in subgroups obtained by dropping increasing percentages of subjects for the whole network. Figure S2: Bootstrapped confidence intervals of estimated edge‐weights for the whole network. Figure S3: Bootstrapped difference test for node strength centrality in the whole network. Figure S4: Bootstrapped difference test for node betweenness centrality in the whole network. Figure S5: Bootstrapped difference test for node closeness centrality in the whole network. Figure S6: Bootstrapped difference test for node expected influence centrality in the whole network. Figure S7: Bootstrapped difference tests between edge‐weights in the whole network. Figure S8: Average correlation between centrality indices of the original whole sample and those estimated in subgroups obtained by dropping increasing percentages of subjects for the ERratio > 1 network. Figure S9: Average correlation between centrality indices of the original whole sample and those estimated in subgroups obtained by dropping increasing percentages of subjects for the ERratio < 1 network. [file PCHJ-15-e70102-s001.zip › Supplementary Figure 2.png]

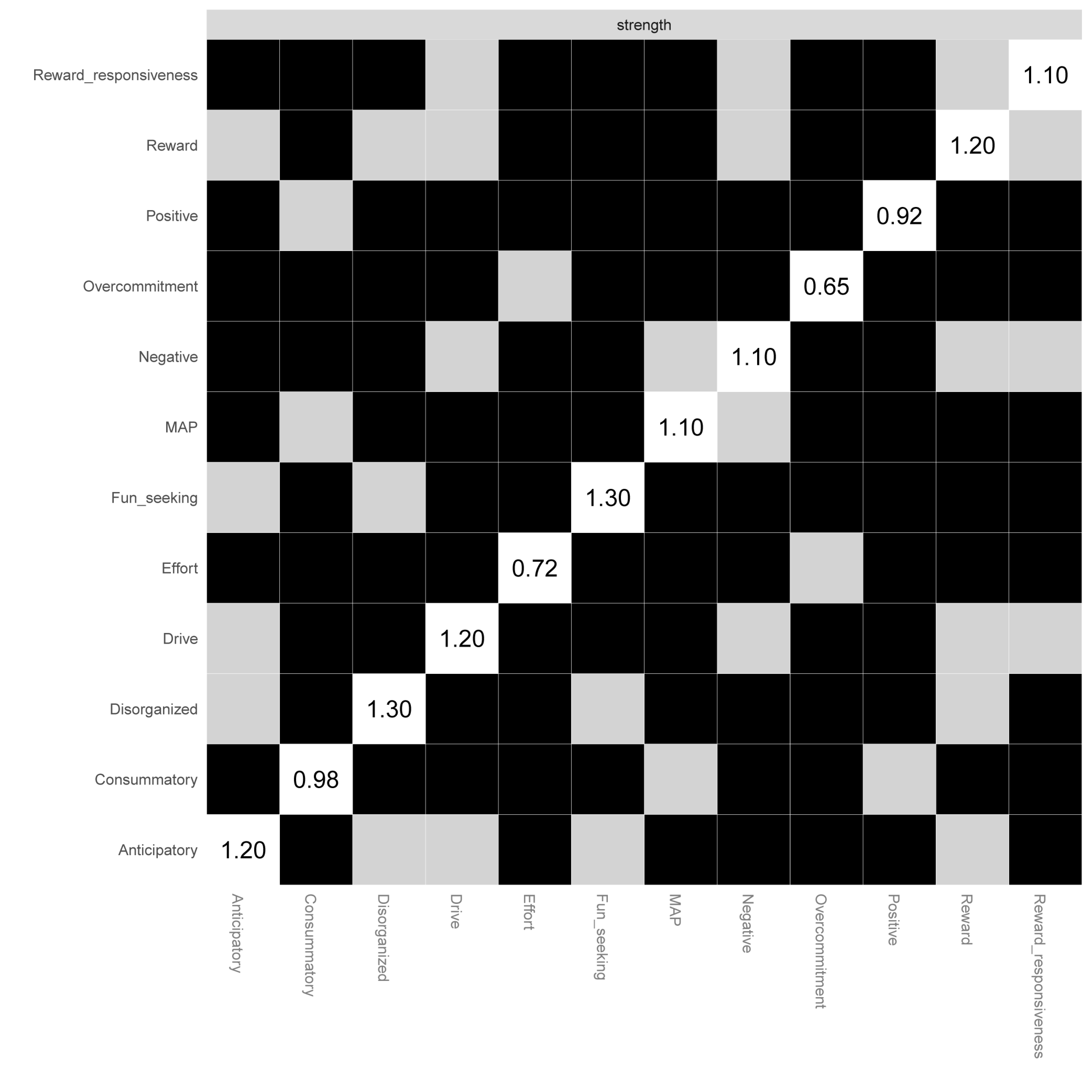

Supplement: Supplementary file 1 — Table S1: Centrality, predictability, expected influence and predictability of nodes in the whole network (n = 6814). Table S2: Normality test of variables in the whole network (n = 6814). Table S3: Zero‐order correlation matrix of variables selected for the whole network (n = 6814). Table S4: Centrality, predictability, expected influence, and predictability of nodes in the ERratio < 1 network (n = 3673). Table S5: Centrality, predictability, expected influence, and predictability of nodes in the ERratio > 1 network (n = 3062). Figure S1: Average correlation between centrality indices of the original whole sample and those estimated in subgroups obtained by dropping increasing percentages of subjects for the whole network. Figure S2: Bootstrapped confidence intervals of estimated edge‐weights for the whole network. Figure S3: Bootstrapped difference test for node strength centrality in the whole network. Figure S4: Bootstrapped difference test for node betweenness centrality in the whole network. Figure S5: Bootstrapped difference test for node closeness centrality in the whole network. Figure S6: Bootstrapped difference test for node expected influence centrality in the whole network. Figure S7: Bootstrapped difference tests between edge‐weights in the whole network. Figure S8: Average correlation between centrality indices of the original whole sample and those estimated in subgroups obtained by dropping increasing percentages of subjects for the ERratio > 1 network. Figure S9: Average correlation between centrality indices of the original whole sample and those estimated in subgroups obtained by dropping increasing percentages of subjects for the ERratio < 1 network. [file PCHJ-15-e70102-s001.zip › Supplementary Figure 3.png]

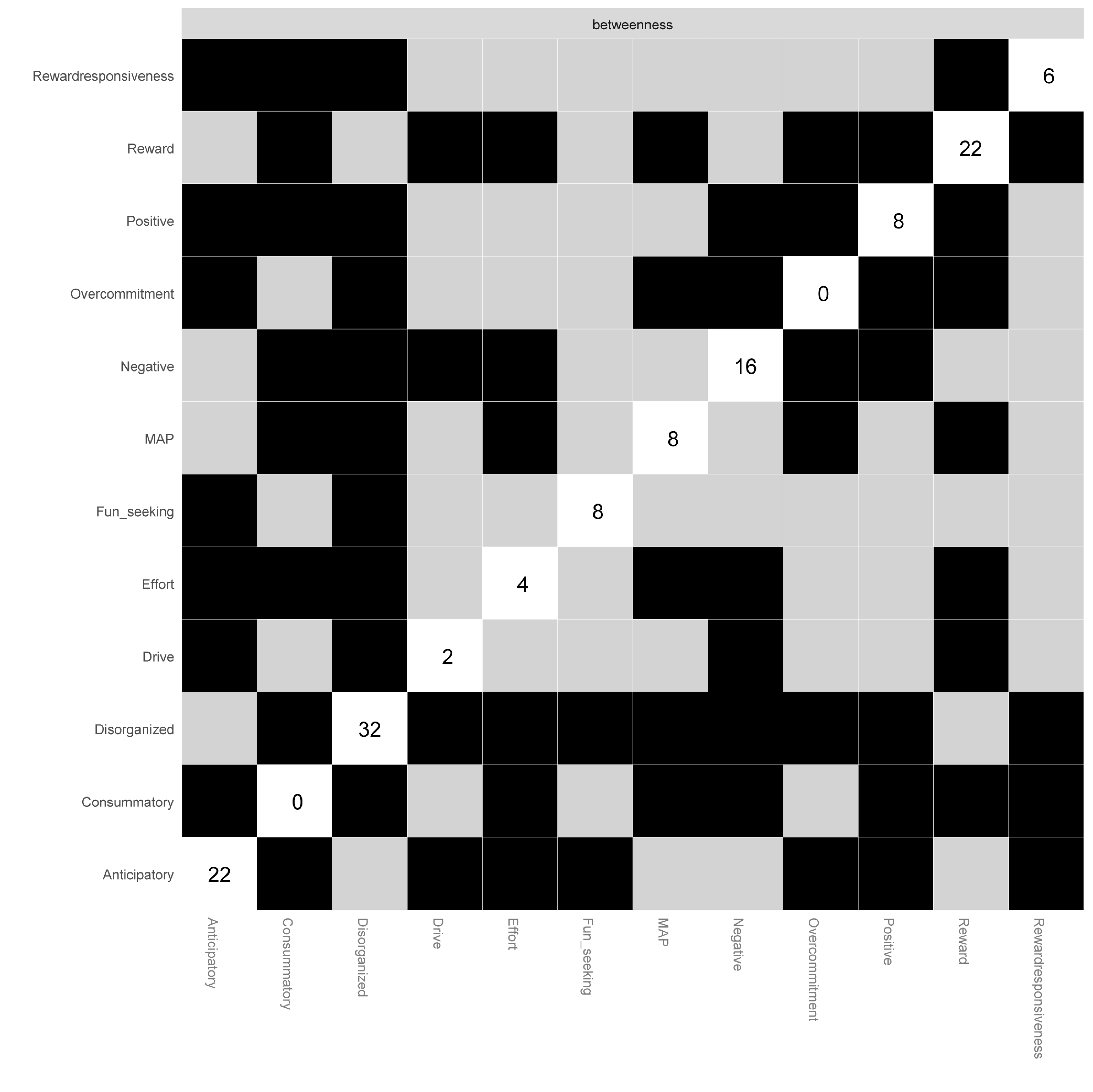

Supplement: Supplementary file 1 — Table S1: Centrality, predictability, expected influence and predictability of nodes in the whole network (n = 6814). Table S2: Normality test of variables in the whole network (n = 6814). Table S3: Zero‐order correlation matrix of variables selected for the whole network (n = 6814). Table S4: Centrality, predictability, expected influence, and predictability of nodes in the ERratio < 1 network (n = 3673). Table S5: Centrality, predictability, expected influence, and predictability of nodes in the ERratio > 1 network (n = 3062). Figure S1: Average correlation between centrality indices of the original whole sample and those estimated in subgroups obtained by dropping increasing percentages of subjects for the whole network. Figure S2: Bootstrapped confidence intervals of estimated edge‐weights for the whole network. Figure S3: Bootstrapped difference test for node strength centrality in the whole network. Figure S4: Bootstrapped difference test for node betweenness centrality in the whole network. Figure S5: Bootstrapped difference test for node closeness centrality in the whole network. Figure S6: Bootstrapped difference test for node expected influence centrality in the whole network. Figure S7: Bootstrapped difference tests between edge‐weights in the whole network. Figure S8: Average correlation between centrality indices of the original whole sample and those estimated in subgroups obtained by dropping increasing percentages of subjects for the ERratio > 1 network. Figure S9: Average correlation between centrality indices of the original whole sample and those estimated in subgroups obtained by dropping increasing percentages of subjects for the ERratio < 1 network. [file PCHJ-15-e70102-s001.zip › Supplementary Figure 4.png]

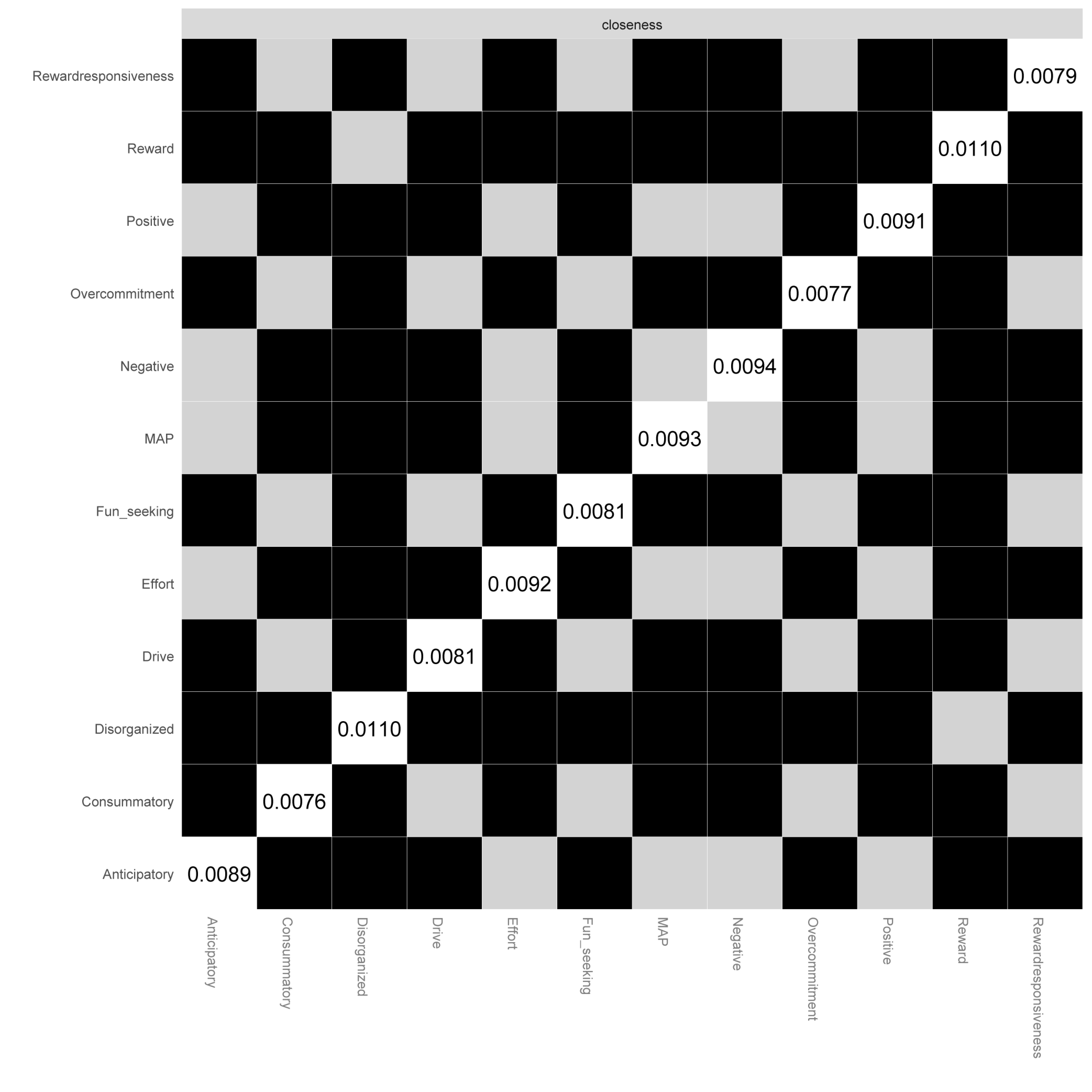

Supplement: Supplementary file 1 — Table S1: Centrality, predictability, expected influence and predictability of nodes in the whole network (n = 6814). Table S2: Normality test of variables in the whole network (n = 6814). Table S3: Zero‐order correlation matrix of variables selected for the whole network (n = 6814). Table S4: Centrality, predictability, expected influence, and predictability of nodes in the ERratio < 1 network (n = 3673). Table S5: Centrality, predictability, expected influence, and predictability of nodes in the ERratio > 1 network (n = 3062). Figure S1: Average correlation between centrality indices of the original whole sample and those estimated in subgroups obtained by dropping increasing percentages of subjects for the whole network. Figure S2: Bootstrapped confidence intervals of estimated edge‐weights for the whole network. Figure S3: Bootstrapped difference test for node strength centrality in the whole network. Figure S4: Bootstrapped difference test for node betweenness centrality in the whole network. Figure S5: Bootstrapped difference test for node closeness centrality in the whole network. Figure S6: Bootstrapped difference test for node expected influence centrality in the whole network. Figure S7: Bootstrapped difference tests between edge‐weights in the whole network. Figure S8: Average correlation between centrality indices of the original whole sample and those estimated in subgroups obtained by dropping increasing percentages of subjects for the ERratio > 1 network. Figure S9: Average correlation between centrality indices of the original whole sample and those estimated in subgroups obtained by dropping increasing percentages of subjects for the ERratio < 1 network. [file PCHJ-15-e70102-s001.zip › Supplementary Figure 5.png]

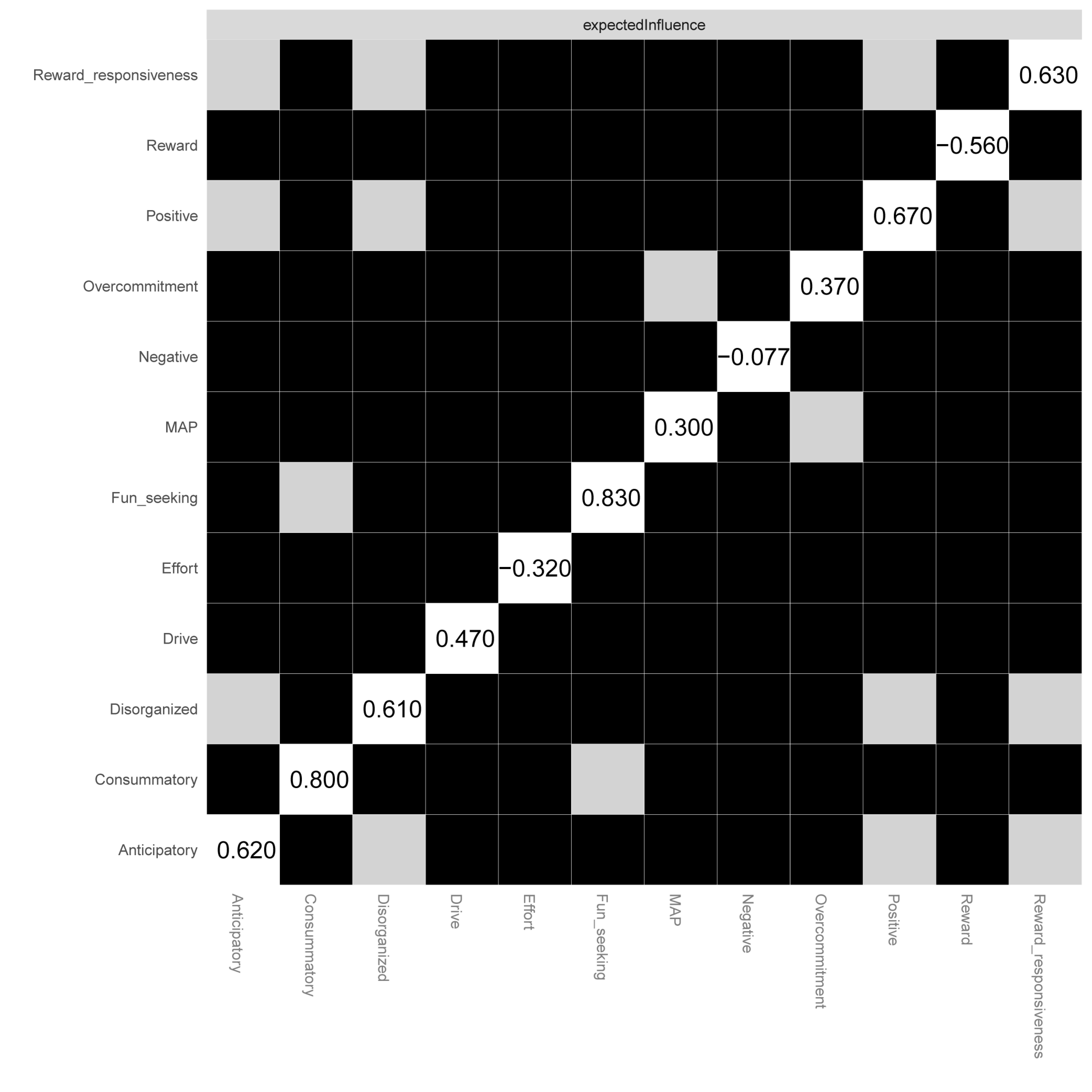

Supplement: Supplementary file 1 — Table S1: Centrality, predictability, expected influence and predictability of nodes in the whole network (n = 6814). Table S2: Normality test of variables in the whole network (n = 6814). Table S3: Zero‐order correlation matrix of variables selected for the whole network (n = 6814). Table S4: Centrality, predictability, expected influence, and predictability of nodes in the ERratio < 1 network (n = 3673). Table S5: Centrality, predictability, expected influence, and predictability of nodes in the ERratio > 1 network (n = 3062). Figure S1: Average correlation between centrality indices of the original whole sample and those estimated in subgroups obtained by dropping increasing percentages of subjects for the whole network. Figure S2: Bootstrapped confidence intervals of estimated edge‐weights for the whole network. Figure S3: Bootstrapped difference test for node strength centrality in the whole network. Figure S4: Bootstrapped difference test for node betweenness centrality in the whole network. Figure S5: Bootstrapped difference test for node closeness centrality in the whole network. Figure S6: Bootstrapped difference test for node expected influence centrality in the whole network. Figure S7: Bootstrapped difference tests between edge‐weights in the whole network. Figure S8: Average correlation between centrality indices of the original whole sample and those estimated in subgroups obtained by dropping increasing percentages of subjects for the ERratio > 1 network. Figure S9: Average correlation between centrality indices of the original whole sample and those estimated in subgroups obtained by dropping increasing percentages of subjects for the ERratio < 1 network. [file PCHJ-15-e70102-s001.zip › Supplementary Figure 6.png]

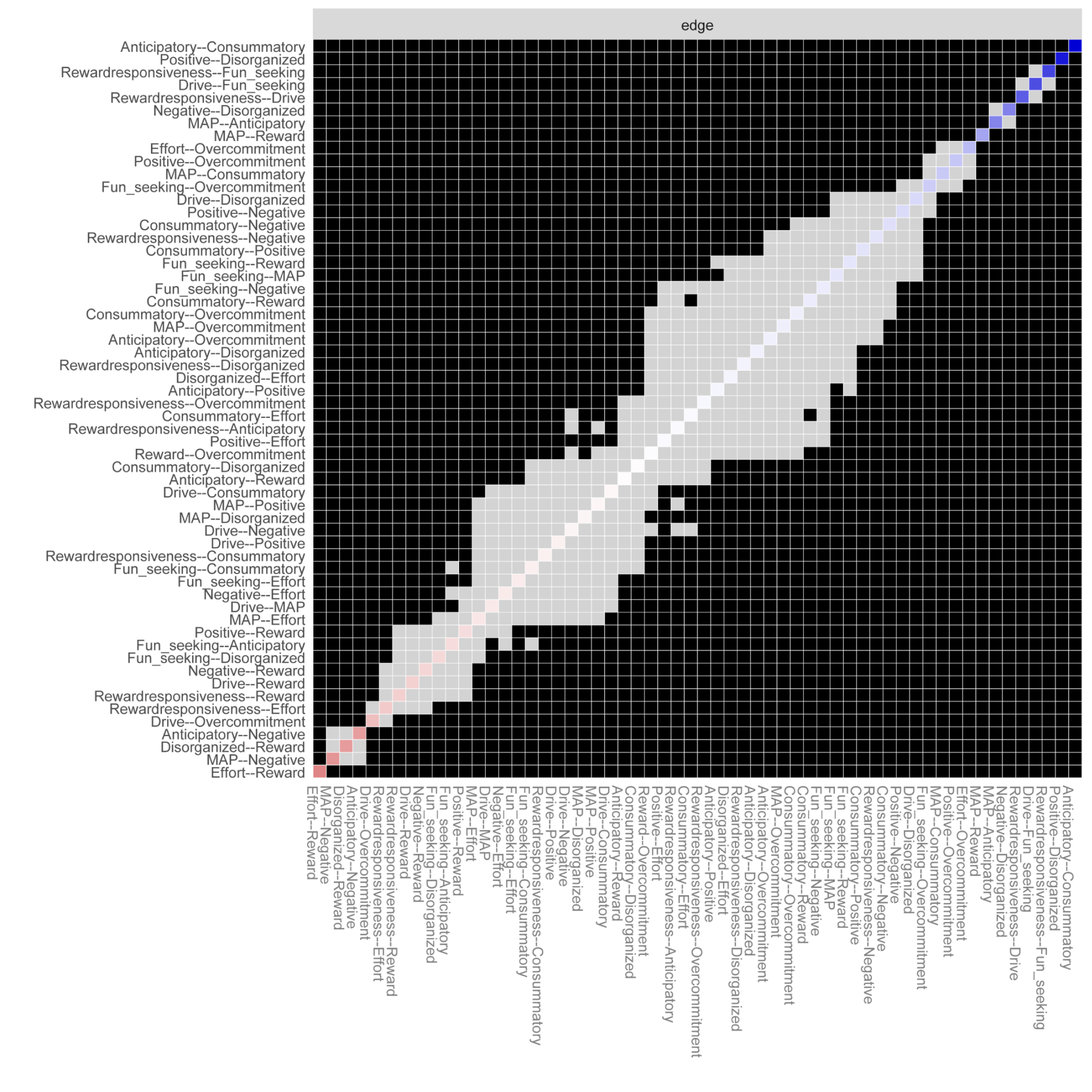

Supplement: Supplementary file 1 — Table S1: Centrality, predictability, expected influence and predictability of nodes in the whole network (n = 6814). Table S2: Normality test of variables in the whole network (n = 6814). Table S3: Zero‐order correlation matrix of variables selected for the whole network (n = 6814). Table S4: Centrality, predictability, expected influence, and predictability of nodes in the ERratio < 1 network (n = 3673). Table S5: Centrality, predictability, expected influence, and predictability of nodes in the ERratio > 1 network (n = 3062). Figure S1: Average correlation between centrality indices of the original whole sample and those estimated in subgroups obtained by dropping increasing percentages of subjects for the whole network. Figure S2: Bootstrapped confidence intervals of estimated edge‐weights for the whole network. Figure S3: Bootstrapped difference test for node strength centrality in the whole network. Figure S4: Bootstrapped difference test for node betweenness centrality in the whole network. Figure S5: Bootstrapped difference test for node closeness centrality in the whole network. Figure S6: Bootstrapped difference test for node expected influence centrality in the whole network. Figure S7: Bootstrapped difference tests between edge‐weights in the whole network. Figure S8: Average correlation between centrality indices of the original whole sample and those estimated in subgroups obtained by dropping increasing percentages of subjects for the ERratio > 1 network. Figure S9: Average correlation between centrality indices of the original whole sample and those estimated in subgroups obtained by dropping increasing percentages of subjects for the ERratio < 1 network. [file PCHJ-15-e70102-s001.zip › Supplementary Figure 7.png]

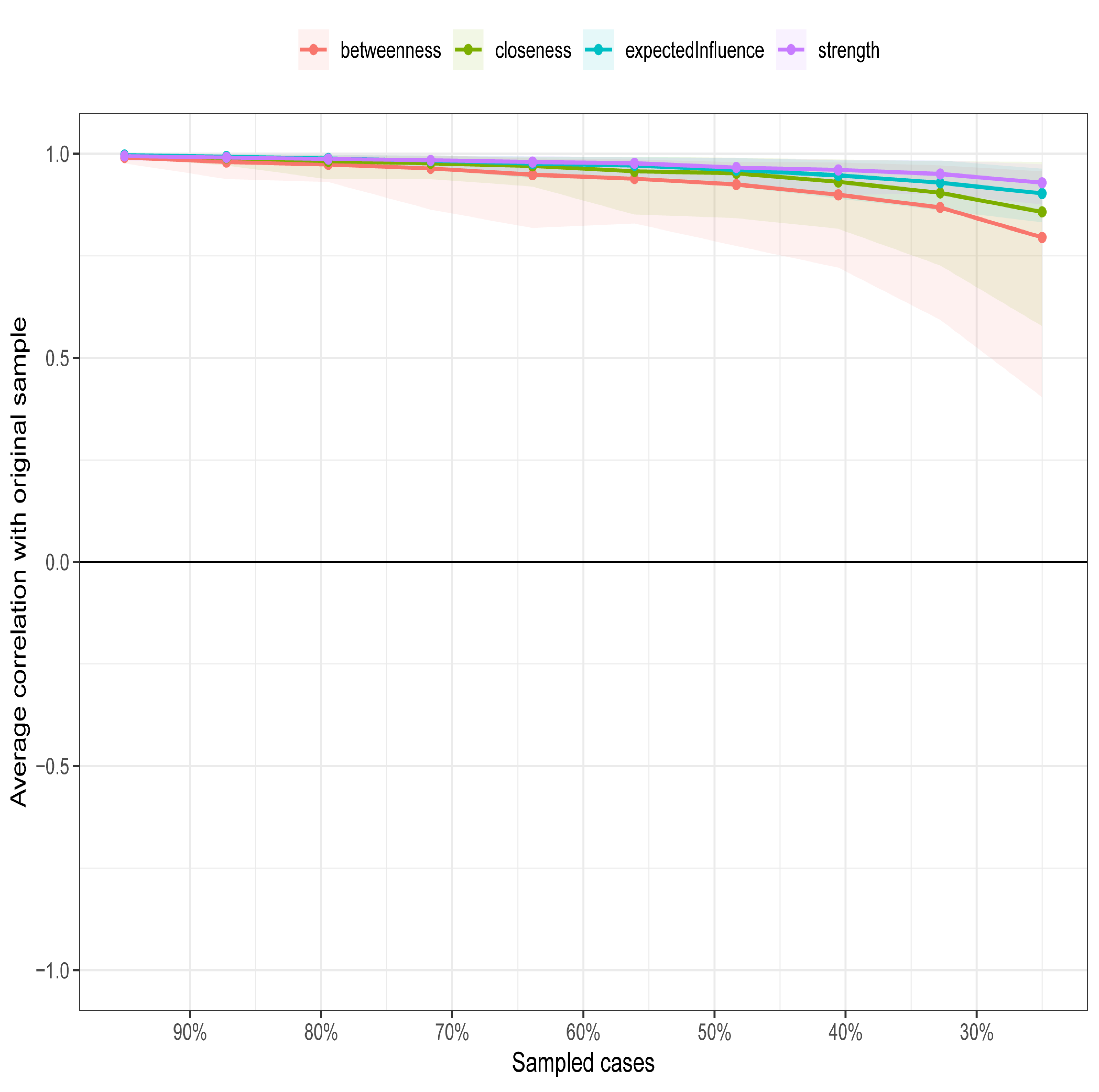

Supplement: Supplementary file 1 — Table S1: Centrality, predictability, expected influence and predictability of nodes in the whole network (n = 6814). Table S2: Normality test of variables in the whole network (n = 6814). Table S3: Zero‐order correlation matrix of variables selected for the whole network (n = 6814). Table S4: Centrality, predictability, expected influence, and predictability of nodes in the ERratio < 1 network (n = 3673). Table S5: Centrality, predictability, expected influence, and predictability of nodes in the ERratio > 1 network (n = 3062). Figure S1: Average correlation between centrality indices of the original whole sample and those estimated in subgroups obtained by dropping increasing percentages of subjects for the whole network. Figure S2: Bootstrapped confidence intervals of estimated edge‐weights for the whole network. Figure S3: Bootstrapped difference test for node strength centrality in the whole network. Figure S4: Bootstrapped difference test for node betweenness centrality in the whole network. Figure S5: Bootstrapped difference test for node closeness centrality in the whole network. Figure S6: Bootstrapped difference test for node expected influence centrality in the whole network. Figure S7: Bootstrapped difference tests between edge‐weights in the whole network. Figure S8: Average correlation between centrality indices of the original whole sample and those estimated in subgroups obtained by dropping increasing percentages of subjects for the ERratio > 1 network. Figure S9: Average correlation between centrality indices of the original whole sample and those estimated in subgroups obtained by dropping increasing percentages of subjects for the ERratio < 1 network. [file PCHJ-15-e70102-s001.zip › Supplementary Figure 8.png]

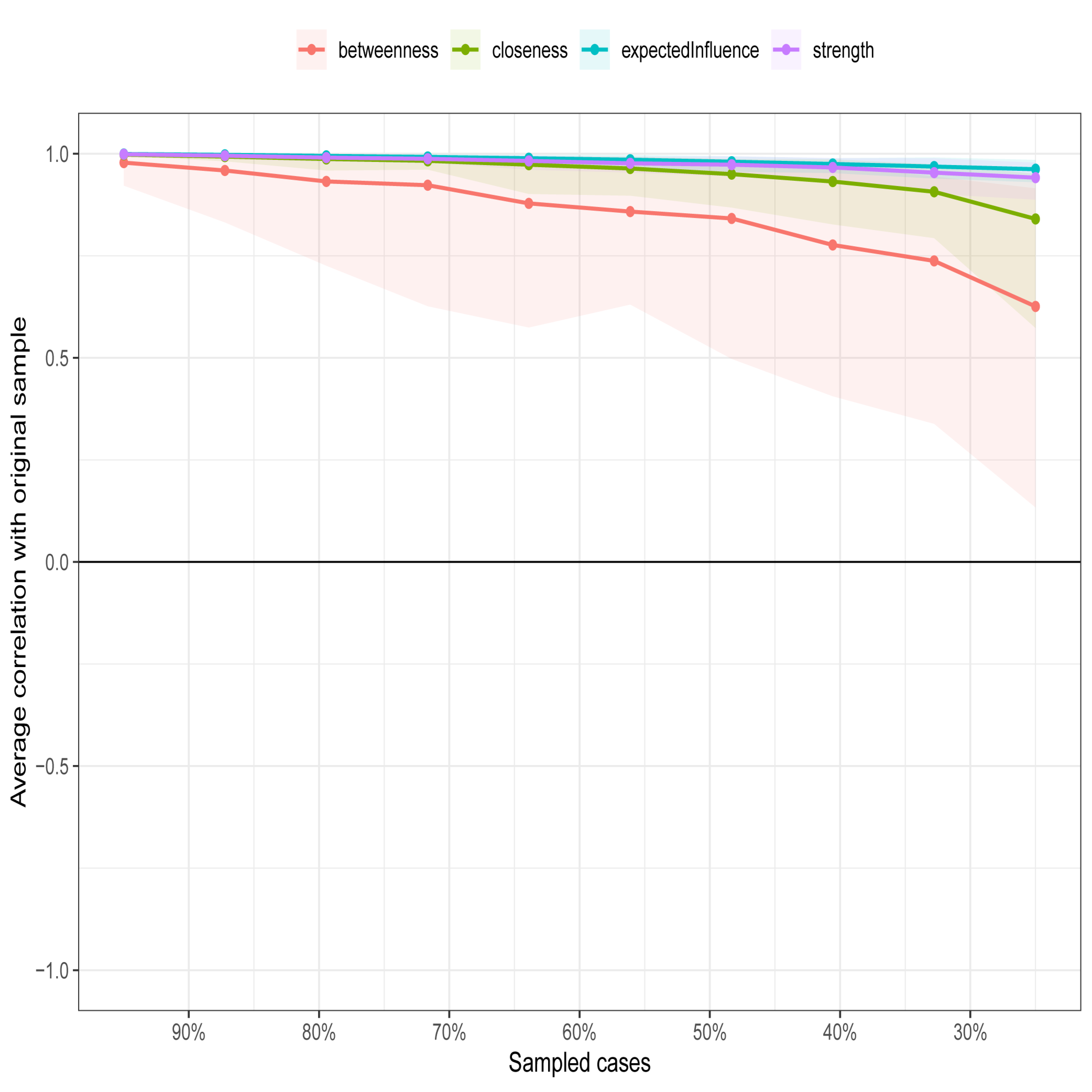

Supplement: Supplementary file 1 — Table S1: Centrality, predictability, expected influence and predictability of nodes in the whole network (n = 6814). Table S2: Normality test of variables in the whole network (n = 6814). Table S3: Zero‐order correlation matrix of variables selected for the whole network (n = 6814). Table S4: Centrality, predictability, expected influence, and predictability of nodes in the ERratio < 1 network (n = 3673). Table S5: Centrality, predictability, expected influence, and predictability of nodes in the ERratio > 1 network (n = 3062). Figure S1: Average correlation between centrality indices of the original whole sample and those estimated in subgroups obtained by dropping increasing percentages of subjects for the whole network. Figure S2: Bootstrapped confidence intervals of estimated edge‐weights for the whole network. Figure S3: Bootstrapped difference test for node strength centrality in the whole network. Figure S4: Bootstrapped difference test for node betweenness centrality in the whole network. Figure S5: Bootstrapped difference test for node closeness centrality in the whole network. Figure S6: Bootstrapped difference test for node expected influence centrality in the whole network. Figure S7: Bootstrapped difference tests between edge‐weights in the whole network. Figure S8: Average correlation between centrality indices of the original whole sample and those estimated in subgroups obtained by dropping increasing percentages of subjects for the ERratio > 1 network. Figure S9: Average correlation between centrality indices of the original whole sample and those estimated in subgroups obtained by dropping increasing percentages of subjects for the ERratio < 1 network. [file PCHJ-15-e70102-s001.zip › Supplementary Figure 9.png]
